# RNAseq reveals extensive metabolic disruptions in the sensitive SF-295 cell line treated with schweinfurthins

**DOI:** 10.1038/s41598-021-04117-7

**Published:** 2022-01-10

**Authors:** J. S. Weissenrieder, J. D. Weissenkampen, J. L. Reed, M. V. Green, C. Zheng, J. D. Neighbors, D. J. Liu, Raymond J. Hohl

**Affiliations:** 1 Department of Physiology, University of Pennsylvania, Philadelphia, PA USA; 2 Department of Medicine, Penn State College of Medicine, Hershey, PA USA; 3 Department of Pharmacology, Penn State College of Medicine, Hershey, PA USA; 4 Penn State Cancer Institute, Penn State College of Medicine, 500 University Drive, Mail Code CH72, Hershey, PA 17033-0850 USA; 5 Department of Pharmacology, The University of Iowa, Iowa City, IA USA; 6 Department of Public Health Sciences, Penn State College of Medicine, Hershey, PA USA; 7 Department of Genetics, University of Pennsylvania, Philadelphia, PA USA

**Keywords:** Cancer, Computational biology and bioinformatics, Drug discovery, Molecular medicine, Mathematics and computing

## Abstract

The schweinfurthin family of natural compounds exhibit a unique and potent differential cytotoxicity against a number of cancer cell lines and may reduce tumor growth in vivo. In some cell lines, such as SF-295 glioma cells, schweinfurthins elicit cytotoxicity at nanomolar concentrations. However, other cell lines, like A549 lung cancer cells, are resistant to schweinfurthin treatment up to micromolar concentrations. At this time, the precise mechanism of action and target for these compounds is unknown. Here, we employ RNA sequencing of cells treated with 50 nM schweinfurthin analog TTI-3066 for 6 and 24 h to elucidate potential mechanisms and pathways which may contribute to schweinfurthin sensitivity and resistance. The data was analyzed via an interaction model to observe differential behaviors between sensitive SF-295 and resistant A549 cell lines. We show that metabolic and stress-response pathways were differentially regulated in the sensitive SF-295 cell line as compared with the resistant A549 cell line. In contrast, A549 cell had significant alterations in response genes involved in translation and protein metabolism. Overall, there was a significant interaction effect for translational proteins, RNA metabolism, protein metabolism, and metabolic genes. Members of the Hedgehog pathway were differentially regulated in the resistant A549 cell line at both early and late time points, suggesting a potential mechanism of resistance. Indeed, when cotreated with the Smoothened inhibitor cyclopamine, A549 cells became more sensitive to schweinfurthin treatment. This study therefore identifies a key interplay with the Hedgehog pathway that modulates sensitivity to the schweinfurthin class of compounds.

## Introduction

Initially isolated from the *Macaranga schweinfurthii* plant in 1987, members of the schweinfurthin family are prenylated stilbenes that show some activity as anticancer agents^1^. Many natural schweinfurthin family members have since been isolated and characterized, including schweinfurthins A-Q^2–5^. Because their extraction is difficult, with characteristically low yields and relative chemical instability, many synthetic schweinfurthin analogs have also been created^6–10^. These have been used to probe structure–activity relationships, improve cytotoxicity, increase stability, and provide fluorescent moieties to enable imaging studies^2,11,12^.

Both natural and synthetic schweinfurthins exhibit a unique cytotoxicity profile in the NCI-60 panel of cell lines, suggesting a novel anticancer mechanism of action^1,2^. Notably, some cell lines are insensitive to schweinfurthins even at high (micromolar) concentrations, whereas others experience profound cytotoxicity at low nanomolar concentrations^2^. This highly differential activity suggests that schweinfurthins may exploit certain sensitivities to elicit cell death in cancer cells, while sparing other cells, possibly including normal tissue. Importantly, sensitive cell lines in the NCI-60 panel represent difficult-to-treat cancers, including triple negative breast cancers, melanomas, and gliomas^2^. Such cancers respond inadequately to current treatments and have poor prognoses for patients, necessitating the development of new therapeutics and novel treatment modalities. Understanding the anticancer mechanism of action of these compounds could therefore inform further drug development and may contribute to improved patient outcomes.

Despite many previous studies, the mechanism of action by which schweinfurthins elicit cytotoxicity is poorly understood, and a molecular target has not yet been identified. Thus far, these compounds have been implicated in: oxysterol binding protein activity, lipid profiles, disruption of the Golgi and endoplasmic reticulum, and cholesterol trafficking, among other processes^2,13–18^. Schweinfurthins have been repeatedly shown to be highly toxic to some cell lines, such as SF-295 glioma cells, but well tolerated by others, including the A549 lung cancer cell line. Although little data is available in vivo, schweinfurthins reduce tumor growth in a Swarm rat chondrosarcoma model^19^ and cooperate with anti-PD-1 therapy to induce tumor regression in a murine B16.F10 xenograft model^15^. However, the mechanisms linking schweinfurthins to ultimate cell death are not known. The low concentrations required in sensitive cell lines suggest that their effects are mediated by a distinct molecular target(s) rather than a general biophysical disruption. Generally, the consensus of the data is that lipid membrane structures may be compromised by schweinfurthin treatment, though the cause of this is not yet clear.

Here, we have considered that analyses of the differential sensitivities of cell lines, such as the well-studied sensitive SF-295 line and the resistant A549 cell line, may provide insights into mechanisms of schweinfurthin action. We hypothesized that genetic or regulatory factors in cell lines contribute to differential schweinfurthin responses in sensitive and resistant cell lines, ultimately leading to survival or cell death outcomes. We therefore analyzed mRNA expression data from the NCI-60 panel of cell lines using sensitivity data from the schweinfurthin analog, methyl-schweinfurthin G (MG) and carried out RNA sequencing experiments to elucidate transcriptional responses to treatment with a novel synthetic schweinfurthin analog, TTI-3066. We carried out experiments at 6 and 24 h timepoints in both the sensitive SF-295 glioma cell line and the resistant A549 lung cancer cell line and compared responses to elucidate early and late transcriptional responses within these representative cell lines, providing key insights into potential mechanisms which contribute to resistance.

## Results

### The NCI-60 panel suggests genes which correlate with sensitivity to schweinfurthins

**Figure 1:**
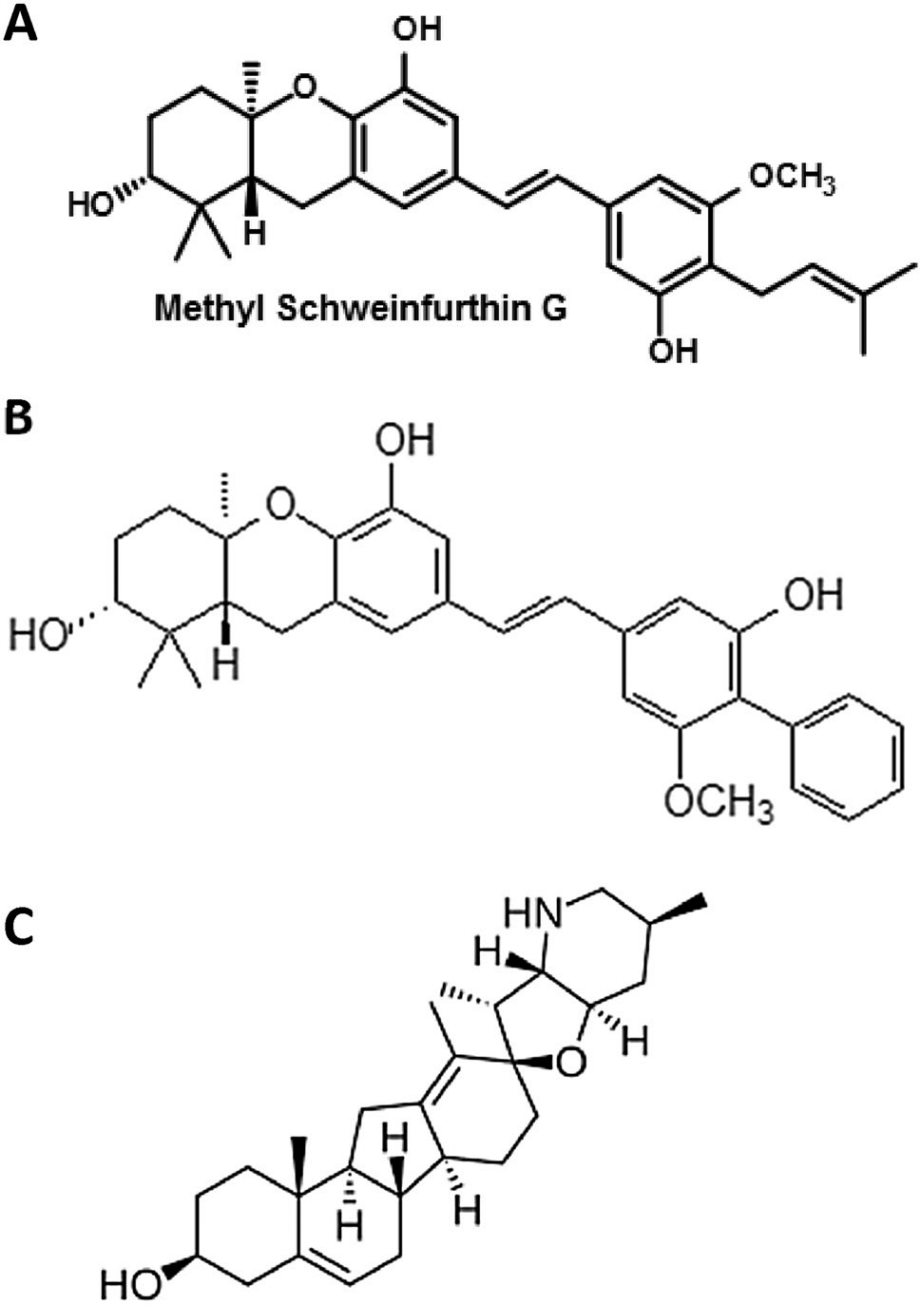
Chemical structures of compounds used. (**A**) Structure of MG, used for NCI-60 panel experiments in Fig. 2 and validations in Supplementary Fig. 5. (**B**) Structure of TTI-3066, used for RNA sequencing experiments and biological validation in Fig. 6. (**C**) Structure of cyclopamine, a selective Hedgehog pathway inhibitor used in Fig. 6 and Supplementary Fig. 5.

**Figure 2:**
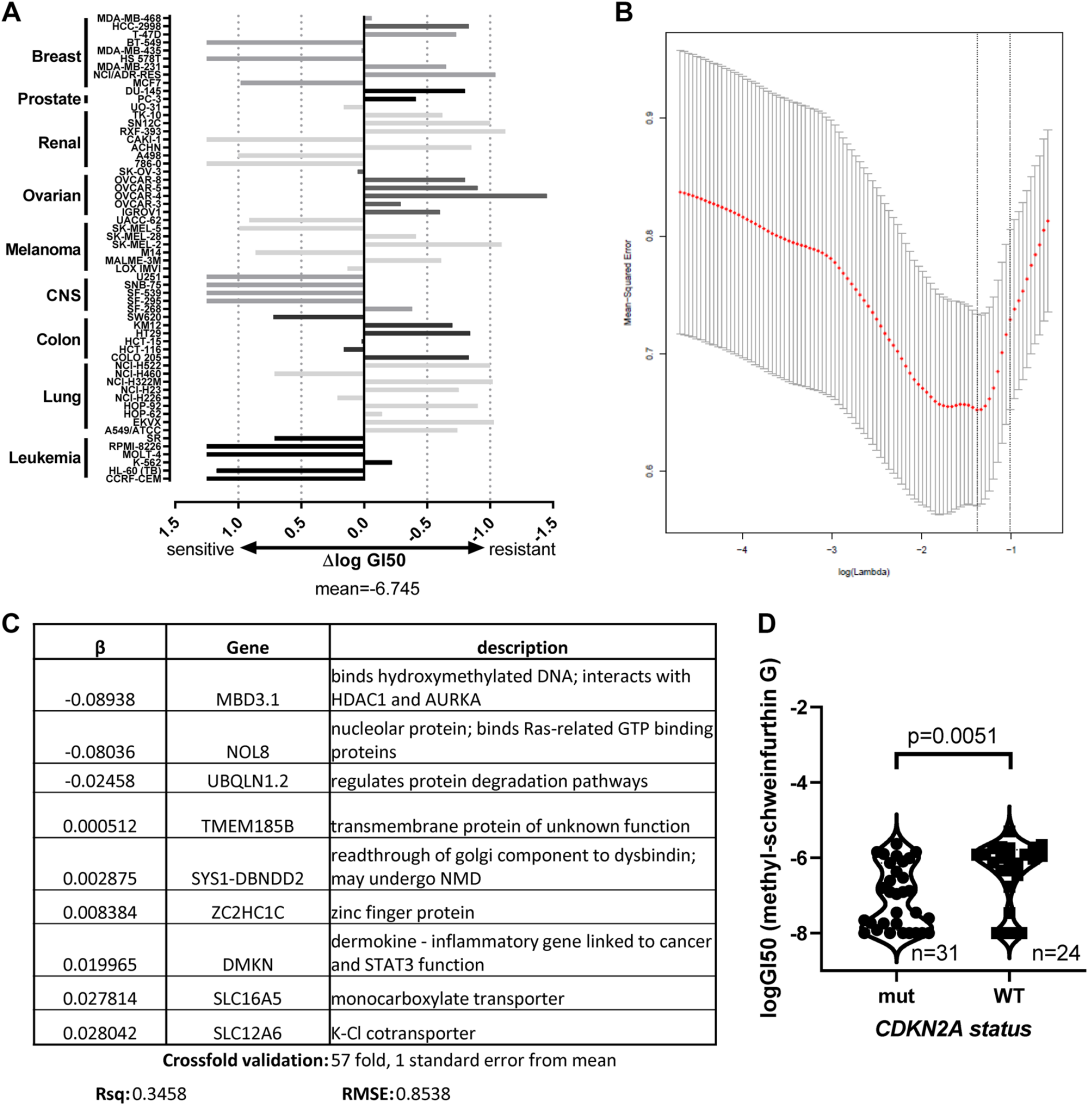
NCI-60 gene expression and schweinfurthin sensitivity. (**A**) Sensitivity of NCI-60 cell lines to MG, expressed as ΔlogGI50(molar) from the mean (− 6.745). Data was obtained through the NCI-60 screening program. (**B**) Lambda vs MSE plot for leave one out crossfold validation of LASSO regression. (**C**) LASSO regression significant genes with β values for leave one out crossfold validation of LASSO regression. Five- and ten-fold cross-validation methods for LASSO regressions were also employed for the same data set and are presented in Supplementary Fig. 1A,B. (**D**) CDKN2A mutations significantly correlate (p = 0.0051) with MG sensitivity as shown by log_10_GI50(M). Other known cancer driver mutations were not significantly correlated with sensitivity and are profiled in Supplementary Fig. 1C.

NCI-60 sensitivity data are available for cell lines treated with MG (Fig. 1A), where sensitivity is described as the half maximal growth inhibitory 50 concentration (GI50) (Fig. 2A). The data are presented as Δlog GI50, which is calculated as the difference between the mean log GI50, − 6.745, and the log GI50 for each cell line. Sensitive cell lines therefore have positive values, shown as bars pointing to the left. LASSO regression was performed with five-, ten-, and 57-fold (leave one out) cross validation paradigms to elucidate genetic factors which may contribute to schweinfurthin sensitivity, utilizing publicly available RNA sequencing data from the NCI. This technique splits the data into partitions, training on all partitions but one, then testing on the partition excluded from the training. To be conservative, we focused on the models where the penalization term, lambda, was maximized while remaining within one standard error from the minimum mean squared error (MSE, Fig. 2B) during cross validation. This results in a model with the minimal number of genes remaining to predict the drug response. The “leave one out” modality proved to be the most parsimonious, with only 9 genes selected in the model at one standard error from the minimum (Fig. 2B,C), whereas fivefold and tenfold cross-validations yielded 14 and 12 genes, respectively (Supplementary Fig. 1A,B). These results indicate that a number of genes may impact cell line sensitivity to schweinfurthins, including those coding for stress response, ion transporter, and plasma membrane proteins. *MBD3.1, NOL8,* and *UBQLN1.2* were associated with schweinfurthin sensitivity, while *TMEM185B, SYS1-DBNDD2, ZCHC1C, DMKN, SLC16A5,* and *SLC12A6* were associated with resistance. Importantly, all gene lists coincided well with each other, with each model including genes from the more parsimonious models before it.

Due to the small sample size, complexity of the cancer state, and Bonferroni corrections, a simple two-variable linear model analysis was unable to identify significant relationships between MG sensitivity and mRNA expression of any single gene (Supplementary Table 1). This method is also highly susceptible to confounding by cell line type factors, since only one gene is included in each model. Indeed, the top two genes in this screen, *OXTR* and *AMPD2*, are most highly expressed in brain and secretory tissues, which tend to be highly sensitive to schweinfurthin treatment (Fig. 1A).

We also compared MG sensitivity data to a panel of known cancer driver mutations present in cancer cell lines within the NCI-60 panel^20^. To avoid confounders, we only analyzed cell line and mutation data in which at least five cell lines in the NCI-60 panel were altered (over 7.5%), data were available for both mutation status and GI50 for MG, and the mutated gene was identified in at least two tumor types. These requirements allowed for a reduction in the potential for confounding by tissue-type effects or insufficient cell line representation and resulted in the inclusion of 8 genes with driver mutations in our analysis (Supplementary Fig. 1C). While this analysis was limited by the relatively small number of cell lines for which we had GI50 data, we observed a significant association between *CDKN2A* mutation status and schweinfurthin sensitivity (n = 31 cell lines mutated, β = − 0.92221, p = 0.0051, Fig. 2D). This result may be significant because *CDKN2A* is commonly mutated in a number of difficult-to-treat cancers, such as glioblastomas and pancreatic ductal adenocarcinomas. Such mutations are associated with a loss of function in the p16 and p14ARF proteins it encodes, permitting unchecked progression through the cell cycle and leading to uncontrolled proliferation.

### RNAseq describes differential gene expression in untreated SF-295 and A549 cells

**Figure 3:**
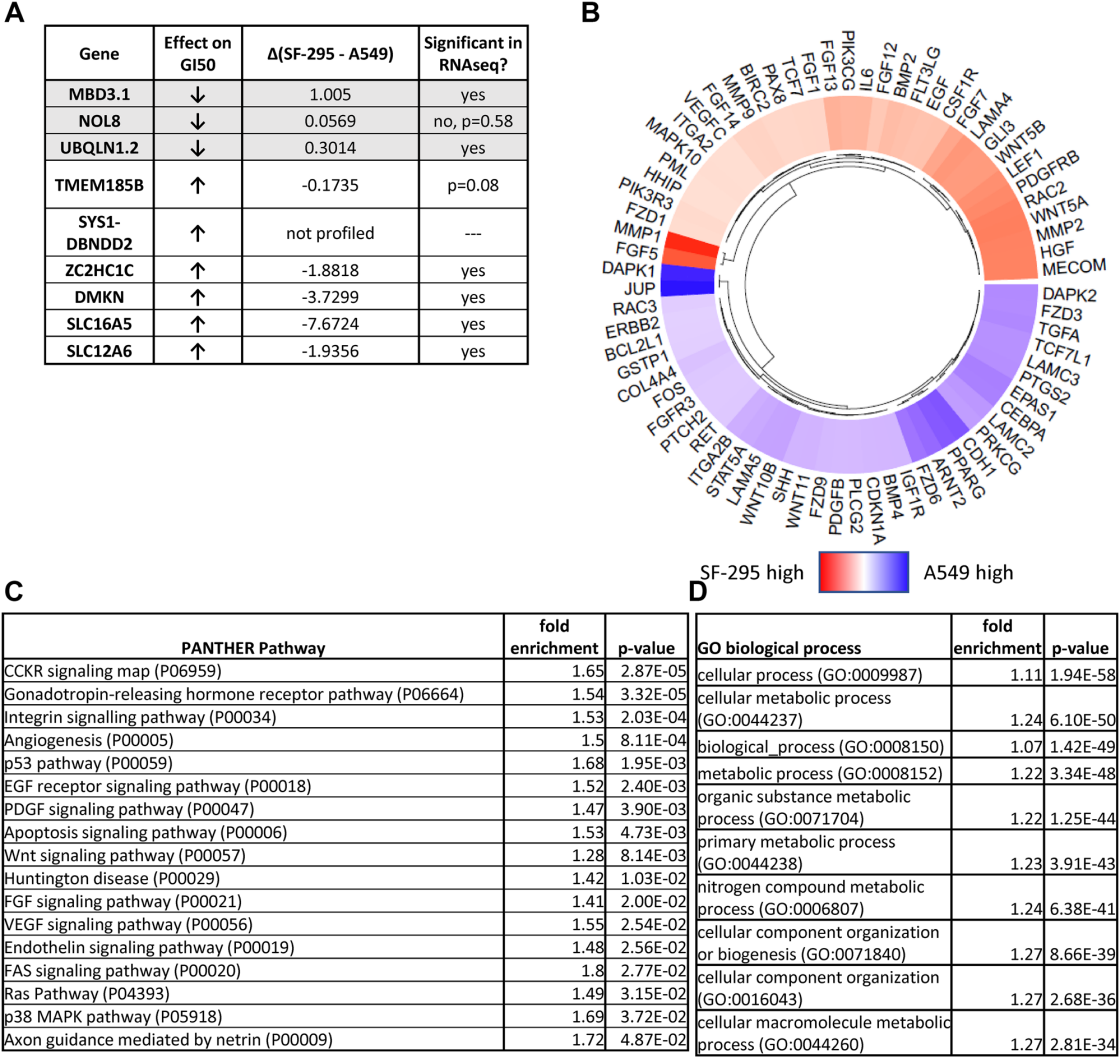
Sensitive SF-295 glioma and resistant A549 lung cancer cells have different gene expression profiles. Untreated SF-295 and A549 cells were analyzed by RNA sequencing. (**A**) Genes identified in the LASSO regression of Fig. 1B compared to RNAseq analysis. (Five-and ten-fold cross-validation comparisons are identified in Supplementary Fig. 2). Most genes identified via LASSO had significantly different expression levels in SF-295 and A549. (**B**) heat map of significantly differentially expressed genes in untreated SF-295 and A549 cells. Genes present in the GSEA geneset “KEGG Pathways in Cancer” with |log fold difference|> 2 were included in the heatmap. Full data is provided in Supplementary Tables 2–3. (**C**) Gene set enrichment analysis of untreated SF-295 and A549 cells with PANTHER pathways was carried out using significantly differentially expressed (adj. p value < 0.05) genes. (**D**) Top hits for GO biological process analysis at 24 h; further reactome and GO pathway analyses are included in Supplementary Table 4.

To develop an understanding of mRNA responses in sensitive and resistant cell lines, we employed RNAseq to compare treated and untreated cell lines after 6 or 24 h of incubation with TTI-3066, a synthetic schweinfurthin analog (Supplementary Table 2). For these experiments, the commonly used models of sensitivity, SF-295 glioblastoma cells, and of resistance, A549 lung cancer cells, were used. Both cell lines are included in the NCI-60 panel and were thus included in the analyses discussed above (of note, both of these cell lines contain mutant *CDKN2A*). Cells were treated with 50 nM TTI-3066 (Fig. 1B), a potent schweinfurthin analog, or vehicle (DMSO) for 6 or 24 h before harvest and RNAseq analysis. The time and concentration were selected to maximize cell line survival and transcriptional responses. The data were compared between untreated groups of both cell lines (Fig. 3A,B, Supplementary Fig. 3) to look at baseline differences which may contribute to sensitivity. Importantly, these data recapitulated much of those in the model generated above, with significant differences between the sensitive SF-295 and insensitive A549 cell lines at baseline including 8 of 14 genes from the fivefold model, with an additional gene nearing significance (Supplementary Fig. 2). Two of the genes identified in Fig. 2 were not profiled in our RNAseq experiments (*TMEM31, SYS1-DBNDD2*). For the most parsimonious model (leave-one-out analysis at the distance of one standard error), 6 of 9 genes were significantly different between SF-295 and A549, with one not profiled and one approaching significance (Fig. 3A). This provides a level of technical verification of the publicly-available dataset used for Fig. 2. Given that the representative cell lines are derived from different types of cancer, it is not surprising that they have very different mRNA expression signatures in cancer-related genes identified from the KEGG cancer pathways gene lists^21–23^ (Fig. 3B). The differences are also reflected in a number of different PANTHER signaling pathways (Fig. 3C), including angiogenesis, Wnt, Ras, p53, and integrin signaling pathways. GO biological process profiling revealed extensive differences in metabolic processes between these cell lines at baseline, notably including genes included in the TCA (tricarboxylic acid) cycle and in the electron transport chain (Fig. 3D, Supplementary Table 4). In general, TCA cycle genes were more highly expressed in the SF-295 cell line, including *PDHA1, ACO1, CS, OGDH, SDHB, SDHD, MDH2,* and *FH*. In the oxidative phosphorylation pathway, 57 genes were significantly differently expressed at baseline, with subunits of complex I (NADH ubiquinone oxidoreductase, including *NDUFA5, NDUFA7, NDUFA8, NDUFB2, NDUFB4, NDUFB5, NDUFB7,* and *NDUFB8*), complex II (*SDHD*), complex III (*UQCRC1, UQCRQ, UQCR10,* and *UQCR11*), and ATP synthase (*ATP5F1A, ATP5F1D, ATP5ME, ATP5MF, ATP5MG, ATP5PB, ATP5PD, ATP5PF, ATP5PO*), suggesting that SF-295 cells are more reliant on these pathways in the absence of applied stressors. However, A549 cells had higher expression of a number of key genes in these pathways. These include *IDH1* and *IDH2*, which may provide a method for the cells to balance NADP+/NADPH levels between the mitochondrial and cytosolic compartments. Similarly, A549 cells have a higher basal expression of *SLC25A23,* which encodes for a mitochondrial membrane solute carrier which may facilitate the transfer of metabolites between compartments. A number of complex IV genes (*COX15, COX5A, COX6B1, COX7A2L, COX8A*) are also more highly expressed in A549.

### RNAseq reveals gene disruptions and pathway involvement in TTI-3066 sensitivity

To elucidate changes in gene expression between sensitive SF-295 and insensitive A549 cell lines in response to schweinfurthin treatment, we treated subconfluent flasks of cells with 50 nM TTI-3066 (Fig. 1B), a novel, highly potent and selective schweinfurthin analog, for 6 h or 24 h. These time points allowed for the discernment of early transcriptional responses at 6 h, followed by slower stress responses at 24 h. We used a relatively low concentration of 50 nM to ensure sufficient cell survival at 24 h to assay mRNA expression, while maximizing cellular responses, in the highly sensitive SF-295 cell line.

**Figure 4:**
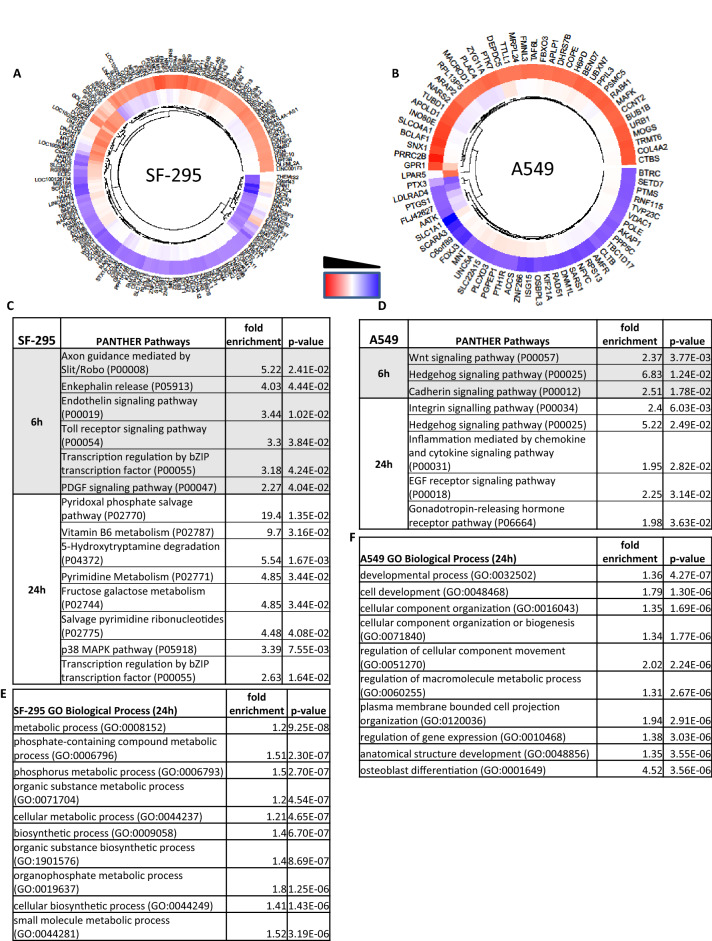
RNAseq of SF-295 and A549 cells treated with 50 nM TTI-3066 for 6 h or 24 h. Data is expressed as log_10_(treated–untreated). All heatmaps were generated with |log_10_(treated–untreated)|> 2, with 6 h data represented as the outside of the ring and 24 h data as the inside of the ring. Heatmaps of significantly altered genes in SF-295 (**A**) and A549 (**B**) are presented. Gene enrichment analysis for PANTHER pathways are included for SF-295 (**C**) and A549 (**D**) at 6 h and 24 h. Reactome and GO pathway analysis are included in supplemental tables. Principal components plots for 6 h (Supplementary Fig. 3A) and 24 h (Supplementary Fig. 3C) are included. At 6 h (Supplementary Fig. 3B) and 24 h (Supplementary Fig. 3D), some genes were differentially regulated similarly between SF-295 and A549. GO biological process top hits at 24 h for SF-295 (**E**) and A549 (**F**) are shown, with further GO and Reactome pathway analysis included in Supplementary Table 4.

Gene expression response to treatment varied greatly between the two cell lines (Fig. 4A,B). In SF-295 cells, enkephalin release, axon guidance, bZIP transcriptional regulation, and signaling pathways such as PDGF and Toll receptor signaling were activated at 6 h (Fig. 4C). By 24 h, the cells showed signs of profound metabolic dysregulation, with alterations in salvage pathways (*PNPO, PDXK)*, alternative carbon source pathways (*KHK, HKDC1*), and p38 MAPK signaling (*CREB1, MAPK11*, and *SRF*); bZIP transcriptional regulators were also significantly altered (Fig. 4C). The bZIP transcription factors included *CREB, Fos/jun,* and *ATF*s involved in inflammatory stress responses. Expression of metabolic pathway genes, including *IDH2, OGDH* (adj. p = 0.051), and *DHCR24*, generally increased in response to TTI-3066 treatment, leading to significant changes in GO biological process pathway enrichment for those involved in metabolism, biosynthesis, and phosphate-containing organic substance pathways (Fig. 4E, Supplementary Table 4). Such findings, in light of the known high sensitivity of this cell line to schweinfurthins, are consistent with a metabolic crisis in which cells attempt to compensate by upregulation of key genes.

In contrast, A549 cells responded to schweinfurthin treatment by altering expression levels of genes in pathways associated with cell growth and differentiation. At 6 h, Wnt, hedgehog, and Cadherin pathway changes were evident, but metabolic genes generally remained unchanged (Fig. 4D). By 24 h, integrin signaling, EGF receptor signaling, and inflammatory pathway gene expression were altered. Whereas SF-295 cells increased the expression of genes included in many metabolic pathways under TTI-3066 treatment, A549 cells generally increased the expression of genes involved in structural regulation, development, and differentiation, as shown by GO biological process pathway enrichment analysis (Fig. 4F, Supplementary Table 4). This suggests a profound difference in drug responses between these two cell lines which may explain their relative sensitivity to this class of compounds. Importantly, Hedgehog pathway genes such as *GLI3* and *SHH* were altered in the resistant A549 cell line at both 6 h and 24 h, but not in SF-295, suggesting a sustained response that may contribute to cellular survival.

Notably, a number of significantly altered genes behaved similarly in both the sensitive SF-295 and resistant A549 cells. At 6 h (Supplementary Fig. 3A), a number of genes showed increased expression, including NADH dehydrogenase subunit 1 (*ND1*), exoribonuclease 1 (*ERI1*), and *SLC12A2*, which modulates sodium and chloride transport. Others, such as *PDE4A, LIFR, PRKAA1, PLAA, MNT,* and *STAB2,* had reduced expression in treated cells. By 24 h (Supplementary Fig. 3B), *DNAH12, ST6GALNA3, DHCR24,* and *SRF* had increased expression, whereas *CREB23, APOPT1, CBY1, IL11, ACHE,* and *GCHFR*, among others, had reduced expression. Genes that showed similar changes in both cell lines may be suggestive of the overall general mechanism of this family of compounds. For example, *DHCR24*, 24-dehydrocholesterol reductase, showed increased expression in both cell lines after 24 h of treatment. This finding complements reports suggesting that schweinfurthin treatment impacts lipid homeostasis^2,16,18,24^.

### Interaction effects highlight changes which may confer sensitivity or resistance to schweinfurthin treatment

**Figure 5:**
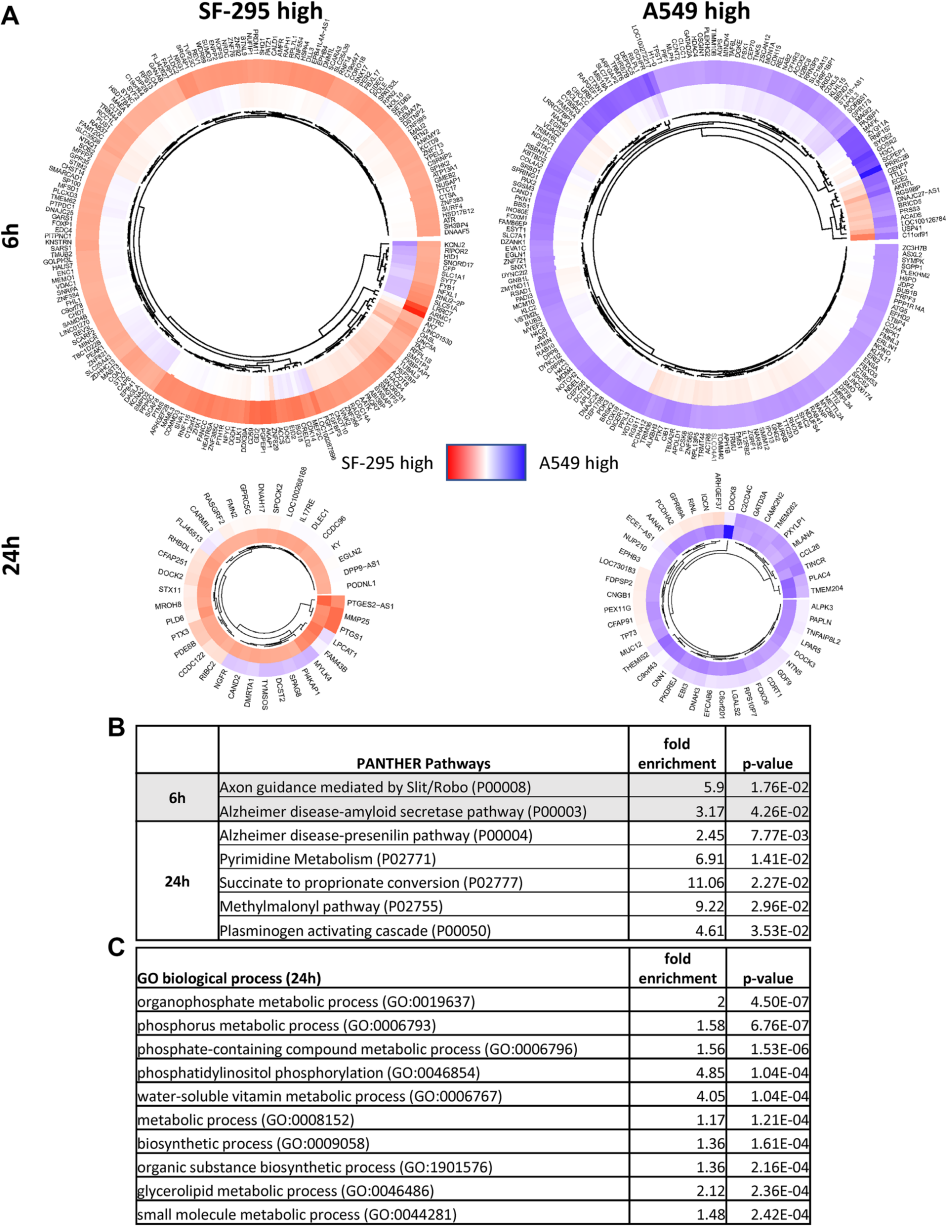
Interaction effects between A549 and SF-295 treated with 50 nM TTI-3066. Interaction effects were calculated as ΔSF-295-ΔA549, and genes with |log_10_(ΔSF-295-ΔA549)|> 2 were included in the heatmap. (**A**) Heatmap of cells treated for 6 h (outer ring) or 24 h (inner ring). (**B**) Gene enrichment analysis for PANTHER pathways are included at 6 h and 24 h. Reactome and GO pathway analysis are included in Supplementary Table 4. (**C**) Representative top pathway hits for the interaction effect at 24 h were determined via GO biological process analysis.

We analyzed interaction effects between SF-295 and A549 responses to TTI-3066 to illuminate potential genes involved in sensitivity and resistance to schweinfurthin treatment at both 6 h and 24 h (Fig. 5A), identifying a number of pathways that were differentially regulated between the two cell lines (Fig. 5B). At 6 h, axon guidance and amyloid secretase pathways showed an interaction effect. While these pathways are not expected to be active in non-neuronal tissues, they speak to general process activation (i.e., cell motility/projection development, metallopeptidase activity, and MAPK signaling). By 24 h, interaction effects were seen in presenilin and plasminogen activation pathways, as well as in the metabolism of pyrimidines and tricarboxylic acid cycle (TCA cycle) related bio-intermediates such as: succinate, proprionate, and methylmalonyl-CoA. GO biological process profiling also indicated significant differences between responses in a number of metabolic and biosynthetic processes, including that of phosphate-containing substrates, vitamins, and glycerolipids (Fig. 5C, Supplementary Table 4). Significantly altered interaction effects were observed in key genes, such as *PSEN1*, *DPYSL5*, *MMP*s, *SERPINE1*, *DGAT1*, *ADCY5*, *PCCB*, *FRZ3*, and *FRZ9*. Metabolic genes showed a positive interaction effect at 6 h (*OGDH*) and 24 h (*ATP5ME, NDUFV1, IDH2,* and *SDHB*), indicating that SF-295 cells were attempting to increase metabolic activity to provide energy. On the other hand, negative interaction effects were observed for *NDUFV1* and *NDUFS4*, suggesting a possible increased need for complex I activity in A549 cells. Such findings suggest that TTI-3066 differentially affects metabolism in these two cell lines, indicating a metabolic crisis and/or possible mitochondrial dysfunction. Importantly, the amyloid secretase and presenilin pathways, as well as phosphatidylinositol phosphorylation, are closely related to calcium signaling and/or inflammatory signals, which are themselves associated with mitochondrial dysfunction^25–27^.

### The Hedgehog pathway interacts with schweinfurthins to regulate sensitivity

**Figure 6:**
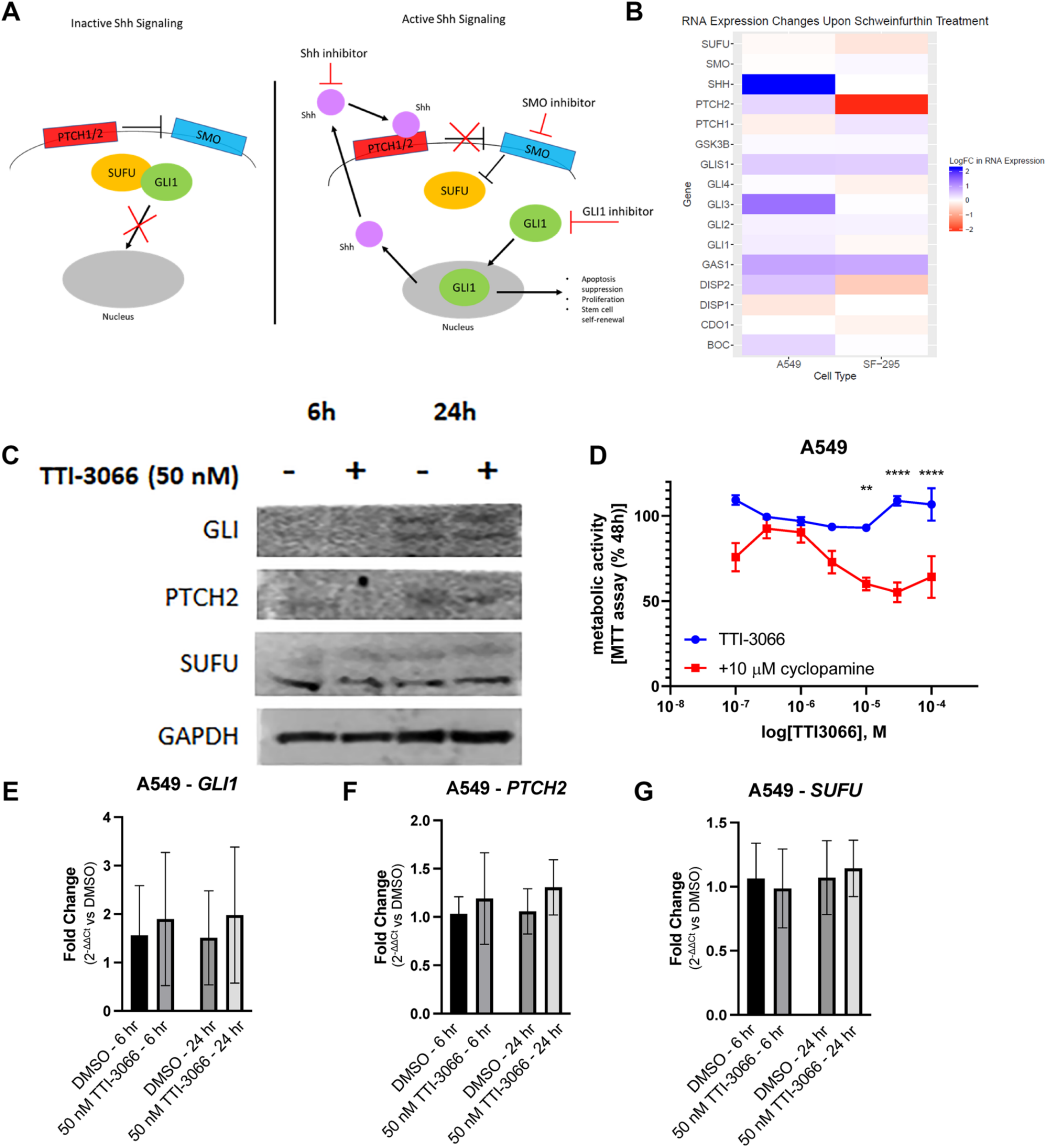
Schweinfurthins affect the hedgehog pathway. (**A**) Schematic of the hedgehog pathway. (**B**) Heatmap of gene expression for selected Hedgehog-related genes at 24 h. (**C**) Western blot of hedgehog pathway genes shows dose and time dependence of hedgehog protein response. (**D**) Compound response was determined via 48 h MTT assay in A549 in the presence or absence of 10 µM cyclopamine, a selective Hedgehog pathway inhibitor. (**E**-**G**) Hedgehog pathway related gene expression changes for *GLI1* (**E**), *PTCH2* (**F**), and *SUFU* (**G**) were investigated via qRT-PCR. Graphs represent fold change with vehicle treatment = 1 following two-way ANOVA with Bonferroni’s correction. GAPDH was used as an internal control gene. Relative gene expression was calculated using the 2^−ΔCt^ method.

One major difference we noted between transcriptional responses to schweinfurthin treatment in sensitive SF-295 and resistant A549 cells was that the resistant cell line has significantly altered expression of Hedgehog pathway genes (Fig. 6A) genes at both 6 h and 24 h, while SF-295 cells did not (Figs. 4C,D, 6B). The Hedgehog pathway is involved in developmental regulation, and it has been repeatedly associated with cancer^28^. Canonically, this pathway is controlled by a ligand/receptor interaction between the Sonic Hedgehog ligand and Patched proteins, which results in disinhibition of Smoothened. This activates a downstream signaling cascade, activating Gli proteins which translocate into the nucleus and act as transcription factors. Significantly, the Hedgehog pathway presents a potentially druggable pathway that appears to be differentially regulated between sensitive and insensitive cancer cell lines. We therefore hypothesized that Hedgehog pathway activity may protect A549 cells against schweinfurthin-induced cytotoxicity, presenting a potential druggable target to enhance schweinfurthin sensitivity. Protein levels of key Hedgehog pathway members were determined via Western blot in A549 cells. Protein levels of Hedgehog pathway members GLI and PTCH2 were slightly reduced after 6 h of treatment with 50 nM TTI-3066, but recovered by 24 h, with no noticeable differences between treated and untreated cells (Fig. 6C). mRNA expression of Hedgehog pathway related genes was confirmed via qRT-PCR in A549 cells. mRNA expression of *GLI1* and *PTCH2* was slightly increased after 6 and 24 h of treatment with 50 nM TTI-3066 (Fig. 6E,F). mRNA expression of *SUFU* was slightly reduced after 6 h of treatment with 50 nM TTI-3066 but recovered by 24 h (Fig. 6G). Differences in the expression of other Hedgehog related genes including *E2F1, PTCH1, PRKACB, IFT88, MTSS1, BNC1, CREBBP,* and *BCL2* trended similarly to the RNA-sequencing results, but differences were not significant (Supplementary Fig. 5). This is consistent with the idea that A549 cells may protect themselves from TTI-3066-induced cytotoxicity in part by increased mRNA expression of pathway members, such as Gli, compensating for protein loss due to cellular stresses. Importantly, the inhibition of Hedgehog pathway signaling with the commonly used Smoothened inhibitor, cyclopamine (10 µM, Figs. 1C, 6A), significantly enhanced cytotoxic response in A549 cells in an MTT assay (Fig. 6D). These results suggest that Hedgehog signaling may contribute to schweinfurthin resistance, and the inhibition of Hedgehog signaling is capable of sensitizing relatively insensitive cancer cells to this potential anticancer agent.

To verify that the effects we have seen may be generalized to the schweinfurthin family, we also observed the effects of MG (Fig. 1A), the earlier generation schweinfurthin analog discussed in Fig. 2, on sensitive SF-295 cells. Inhibition of the Hedgehog pathway with 10 µM cyclopamine enhanced SF-295 cell sensitivity to schweinfurthins (Supplementary Fig. 4A). Indeed, cotreatment with cyclopamine and MG in this cell line suggested that the two may have a synergistic relationship, as shown by isobologram analysis (Supplementary Fig. 4B,C).

## Discussion

Since their initial isolation from extracts of the *Macaranga schweinfurthii* plant, schweinfurthins have generated interest in the cancer therapeutic field due to their novel sensitivity profile in the NCI-60 panel, suggestive of a novel anticancer mechanism. Here, we first utilized publicly available mRNA expression data for the NCI-60 panel to determine genes that may be associated with schweinfurthin resistance or sensitivity via unbiased LASSO regression with crossfold validation. Through this, we identified a group of genes, including *MBD3.1*, *NOL8*, and *UBQLN1.2*, which can identify the sensitivity of cell lines with Rsq = 0.34, despite the small sample size. We also report a significant association between *CDKN2A* mutational status and schweinfurthin sensitivity. Previously, others have studied the schweinfurthin family of compounds via genetic alterations using multivariate modeling of the GDSC (Genomics of Drug Sensitivity in Cancer) panel^29^, where they identified *PLEKHO1*, a negative regulator of PI3K/AKT signaling, as well as *THEM4* and *OSBP* genes, as potential determinants of schweinfurthin response. Importantly, problems have been noted with the use of data sets such as the GDSC and CCLE (Cancer Cell Line Encyclopedia) due to variability between compound sensitivities and, to a lesser degree, gene expression results^30^. This may be due to differences in timing, cell handling, assay type, and other experimental factors between cell lines and treatment paradigms for these large-scale databases which may be attributed to the large scale of these studies. In contrast, the NCI-60 data presented herein is of a reasonable size for comparable treatment, and the compound response data was generated at the same time in all cell lines tested via the same method, enhancing reproducible conditions between cell lines.

Although gene expression may impact compound sensitivity, cancer cell responses to therapy may have more of an effect on sensitivity and resistance than baseline gene expression levels. Therefore, to elucidate the importance of cellular responses to treatment and sensitivity, we generated unbiased gene expression data via RNAseq for representative sensitive (SF-295) and resistant (A549) cell lines at 0, 6, and 24 h of treatment with a highly potent, novel schweinfurthin analog, TTI-3066. Comparisons between the two cell lines suggest that while they both exhibit extensive changes in gene expression, SF-295 cells were generally characterized by salvage and metabolic pathway responses, whereas A549 cells were more associated with changes in growth and differentiation associated pathways. Significant interaction effects were observed in a number of metabolic pathways at 24 h. Importantly, a sustained change in Hedgehog pathway gene expression was seen in TTI-3066 treated resistant cells at both 6 h and 24 h, suggesting a potential mechanism of resistance. Further experiments suggested that inhibition of this pathway with the Smoothened inhibitor, cyclopamine, significantly sensitized A549 cells to TTI-3066. Such findings were generalizable to sensitive cell lines and across multiple schweinfurthin analogs. These results are highly suggestive that Hedgehog pathway signaling provides a resistance mechanism for cancer cells under treatment with schweinfurthins, and thus cotreatment could enhance compound response rates in vitro and in vivo*.* Future studies will be necessary to further define the relationships between Hedgehog pathway signaling and schweinfurthin responses.

This work underscores the validity of using large-scale genetic expression data as a method to identify sensitive cancer cells, mechanisms of drug action, and compound resistance behaviors in an in vitro context, with the potential to expand into in vivo models. Such methods enable unbiased isolation of potential contributor pathways which might not otherwise be selected for study. Such experiments are particularly useful in two areas: (1) the natural products field, where mechanisms are often unclear, and (2) compounds which generate unforeseen resistance responses in cell lines that are not associated with mutational changes. Additionally, gene expression responses may suggest repurposing of other compounds for the treatment of unrelated disorders or predict/explain unforeseen side effect profiles in in vivo models. Accordingly, we expect the availability, scope, and usage of such data sets to increase.

## Methods

### Cell lines and reagents

SF-295 cells were obtained from Addex Bio (San Diego, CA) and cultured in Roswell Park Memorial Institute 1640 (RPMI 1640) medium (Gibco, Waltham, MA) with 10% fetal bovine serum (FBS) added (Hyclone, from ThermoFisher Scientific, Waltham, MA). A549 cells were obtained from ATCC (Manassas, VA) and were cultured in F-12 medium (Gibco, Waltham, MA) with 10% FBS. All cells were maintained at 37 °C and 5% CO_2_ and used within 20 passages.

MG and TTI-3066 was obtained from Terpenoid Therapeutics, Incorporated (Coralville, IA). They were dissolved in dimethylsulfoxide (DMSO) to 100 mM and stored in the dark at − 20 °C before use. This stock was then diluted to 10 mM immediately before dilution to final concentration in complete cell culture media, as above. Cyclopamine (Fig. 1C, SelleckChem, Houston, TX) was dissolved in DMSO to mM before dilution to final concentration in complete cell culture media.

### RNAseq

Cells were plated in T175 flasks and allowed to grow overnight to 80% confluence before treatment. Cells were then treated with 50 nM TTI-3066 in standard growth media, as above, for 6 or 24 h. RNA was harvested on ice with TRIzol (ThermoFisher Scientific, Waltham, MA). RNAseq was performed by the Penn State Hershey Genome Sciences Core (Hershey, PA) to at least 25 million reads per sample on an Illumina NovaSeq 6000 (San Diego, CA). Experiments were carried out in triplicate.

Raw data was aligned to the human genome build 37 using the Rsubread package^31^. Aligned counts were normalized and formatted into log(counts per million (CPM)) and genes were filtered so that only genes with sufficiently large counts remained using the edgeR package as described previously^32^. Once the logCPM was calculated, principal component (PC) analysis was conducted using the limma package^33,34^ to investigate technical batch noise. This technique evaluates the overall agreeableness between replicates to ensure similar expression patterns between replicates. From the PC plots, we determined that one sample in each timepoint was sufficiently different from its technical counterparts to justify its exclusion from the analysis.

The limma package^33–35^ was used to evaluate differences in expression between conditions. We investigated baseline differences between SF-295 and A549, differences upon treatment in expression for each cell line, and the differences in differential expression between the cell lines upon treatment, also considered the interaction as seen in Eq. (1). Moderated t-statistics, moderated F-statistic, and log-odds of differential expression was calculated by the empirical Bayes moderation of standard errors as described by others^36,37^.

$$Interaction\, Contrast = \overbrace {{\left( {SF295_{Treated} - SF295_{Untreated} } \right)}}^{SF295 \,Contrast} - \overbrace {{\left( {A549_{Treated } {-} A549_{Untreated} } \right)}}^{A549 \,Contrast}.$$

To visualize the differences in gene expression between our cell lines, we utilized the circlize package[citation] in R to create heatmaps. Higher gene expression in SF-295 cells was visualized in red, while higher gene expression in A549 cells was shown in blue where absolute value affected intensity of color^38,39^.

### Western blotting

Western blotting was carried out with the Molecular Devices ScanLater system (San Jose, CA), as described previously^40^. Briefly, cells were harvested in RIPA buffer with protease and phosphatase inhibitors and agitated for an hour at 4 °C before protein quantification with a Pierce BCA (bicinchoninic acid) assay. Equal amounts of protein were loaded on precast Bolt 4–12% Bis/Tris gels (Invitrogen) and run at 150 V for 45–60 min in Bolt MOPS(3-(N-morpholino)propanesulfonic acid) buffer before transfer onto a polyvinylidene fluoride membrane (Immobilon-P) in Bolt transfer buffer for 1.5 h at 25 V. Membranes were cut horizontally to enable high quality imaging with multiple antibodies per blot, blocked with 1 × ScanLater buffer, probed with primary antibodies overnight, rinsed with ScanLater wash buffer, and then probed with Eu-labeled (europium labeled) secondary antibodies (goat antimouse: R8205, goat antirabbit: R8204) for 1 h before rinsing membranes and drying them. Dry membranes were imaged with a SpectraMax i3x with a Western blot cartridge (Molecular Devices). Running and transfer buffers were obtained from ThermoFisher Scientific (Waltham, MA), and blots were normalized to loading control, vinculin, and quantified with Licor ImageStudio Lite Version 5.2 software (Lincoln, NE).

### Quantitative real-time PCR

Cells were plated in T175 flasks and allowed to grow overnight to 80% confluence before treatment. Cells were then treated with 50 nM TTI-3066 in standard growth media, as above, for 6 or 24 h. RNA was harvested on ice using RNeasy Mini Kit (Qiagen). Synthesis of cDNA from total RNA was performed following manufacturer’s instruction using superscript III reverse transcriptase (Invitrogen).

Quantitative RT-PCR was performed to confirm expression of hedgehog pathway related genes using the Taqman Array, Human Hedgehog Fast 96-well (Applied Biosystems) system. Briefly the cDNA samples were combined with Taqman Gene Expression Master Mix (Applied Biosystems) and added to the appropriate wells of the plate. The thermal protocols followed were provided by Applied Biosystems and are available upon request. Relative gene expression was calculated using the 2^−ΔCt^ method, ﻿whereas the mean fold change = 2 − (average ∆∆Ct) was assessed using the mean difference in the ∆Ct between the gene and the internal control. Genes assayed include: *E2F1, STIL, SMO, GLI1, SUFU, CSNK1G2, PRKACB, SHH, PTCH1, BTRC, PTCH2, ZIC3, LRP2, IFT88, MTSS1, IFT52, RAB23, CSNK1G1, BNC1, CREBBP, FOXA2, GLI2, CSNK1E, GAS1, MYF5, GSK3B, CSNK1G3, DHH, HHIP, CSNK1D, STK36, DISP2, DISP1, PRKACA, ZIC2, ZIC1, FBXW11, BCL2, GLI3, CSNK1A1, PRKX, PRKY, PRKACG,* and *IHH*. GAPDH was used as an internal control and was not significantly different for 6 and 24 h DMSO and TTI-3066 treatment across three replicates. Standard deviation of GAPDH for 6-h treatment was 0.4371 and 1.558 for DMSO and TTI-3066 respectively. Standard deviation of GAPDH for 24-h treatment was 0.3747 and 0.4102 for DMSO and TTI-3066 respectively. Statistical significance was assessed for all genes assayed using ΔCt values via two-way ANOVA with Bonferroni’s correction with GraphPad Prism 9 (San Diego, CA).

### Data analysis and statistics

#### NCI60 panel variable selection

The National Cancer Institute (NCI) provides a service wherein potential anticancer compounds may be screened against a broad panel of cancer cell lines, known as the NCI-60 panel, to observe anticancer activity in a variety of paradigms. This panel represents a medley of well characterized cell lines of numerous tissue origins, including breast, lung, skin, central nervous system (CNS), renal, prostate, and non-solid tumors. These lines have been genetically characterized by DNA and RNA sequencing. We compared this publicly available RNA expression data to logGI50 (50% growth inhibitory) data from NCI-60 screening (Fig. 2A) to observe potential relationships between gene expression and sensitivity to MG, an early-generation synthetic schweinfurthin analog (Fig. 1A). Previous work has shown that R,R,R isomers of schweinfurthins have similar sensitivity profiles in the NCI-60 panel and similar phenotypic responses in vitro^41^. This analysis employed LASSO (least absolute shrinkage and selection operator) variable selection regression methodologies with cross-validation in the context of publicly available untreated log_2_ expression data from the NCI-60 panel and our results from initial NCI-60 screening. Due to changes in panel composition over time, only 57 cell lines were able to be compared. This regression varies a penalization term, lambda, to filter variables and discerns the performance of models across lambda. LASSO regression was employed because it is highly capable of identifying predictive genes and their relative contributions to the model. Results are presented in Fig. 2 and Supplementary Fig. 1.

#### Pathway analysis

Pathway analysis was carried out using the Gene Ontology Consortium webtool^42–44^ with the Fisher’s exact test. GO biological function, PANTHER pathway, and Reactome Pathways were queried. Interaction, glioma only, lung only, and baseline differences were examined at 6 h and 24 h with an adjusted p-value threshold of 0.05. Full pathway analysis results are included in Supplementary Table 4.

## Supplementary Information

Supplementary Table 1.

Supplementary Table 2.

Supplementary Table 3.

Supplementary Table 4.

Supplementary Legends.

Supplementary Figures.
