# Supplementary material for: RNAseq reveals extensive metabolic disruptions in the sensitive SF-295 cell line treated with schweinfurthins
Source: Sci Rep. 2022 Jan 10;12:359. doi: 10.1038/s41598-021-04117-7 (PMC8748991; doi:10.1038/s41598-021-04117-7)
Supplement: Supplementary file 5 — Supplementary Legends. [file 41598_2021_4117_MOESM5_ESM.pdf]

Supplementary Table 1. Linear modeling of NCI-60 gene expression data and MG sensitivity. Simple, two variable linear models were generated between MG sensitivity (IC50) and each gene in the publicly available RNAseq data for the NCI-60 panel of cell lines (~13,000 genes). No significant correlations were identified after Bonferroni correction. Raw p-values and betas are included for each gene queried.

Supplementary Table 2. RNAseq results from SF-295 and A549 cells treated with 0 or 50 nM TTI-3066 for 6h. RNAseq was performed as described on SF-295 and A549 cells which were treated with 0 nM or 50 nM TTI-3066 for 24h. Genes are identified as gene IDs, gene symbols, and gene names. For each gene, this table contains adjusted p-values and betas for baseline differences (calculated as SF-295 - A549), TTI-3066 associated changes (calculated as treated-untreated) for both SF-295 (glioma) and A549 (lung) cell lunes, and the interaction contrast (calculated as in the methods section).

Supplementary Table 3. RNAseq results from SF-295 and A549 cells treated with 0 or 50 nM TTI-3066 for 24h. RNAseq was performed as described on SF-295 and A549 cells which were treated with 0 nM or 50 nM TTI-3066 for 24h. Genes are identified as gene IDs, gene symbols, and gene names. For each gene, this table contains adjusted p-values and betas for baseline differences (calculated as SF-295 - A549), TTI-3066 associated changes (calculated as treated-untreated) for both SF-295 (glioma) and A549 (lung) cell lunes, and the interaction contrast (calculated as in the methods section).

Supplementary Table 4. Pathway analysis of genes which significantly altered expression under treatment with TTI-3066. Pathway analysis was carried out with the Gene Ontology Consortium webtool as described. Pathway hits for GO biological process, GO molecular function, Reactome, and PANTHER pathways are shown for SF-295 (glioma), A549 (lung), baseline differences (SF-295 - A549) and interaction contrast (as described in methods).
