# Supplementary material for: RNAseq reveals extensive metabolic disruptions in the sensitive SF-295 cell line treated with schweinfurthins
Source: Sci Rep. 2022 Jan 10;12:359. doi: 10.1038/s41598-021-04117-7 (PMC8748991; doi:10.1038/s41598-021-04117-7)
Supplement: Supplementary file 6 — Supplementary Figures. [file 41598_2021_4117_MOESM6_ESM.pptx]

## Slide 1
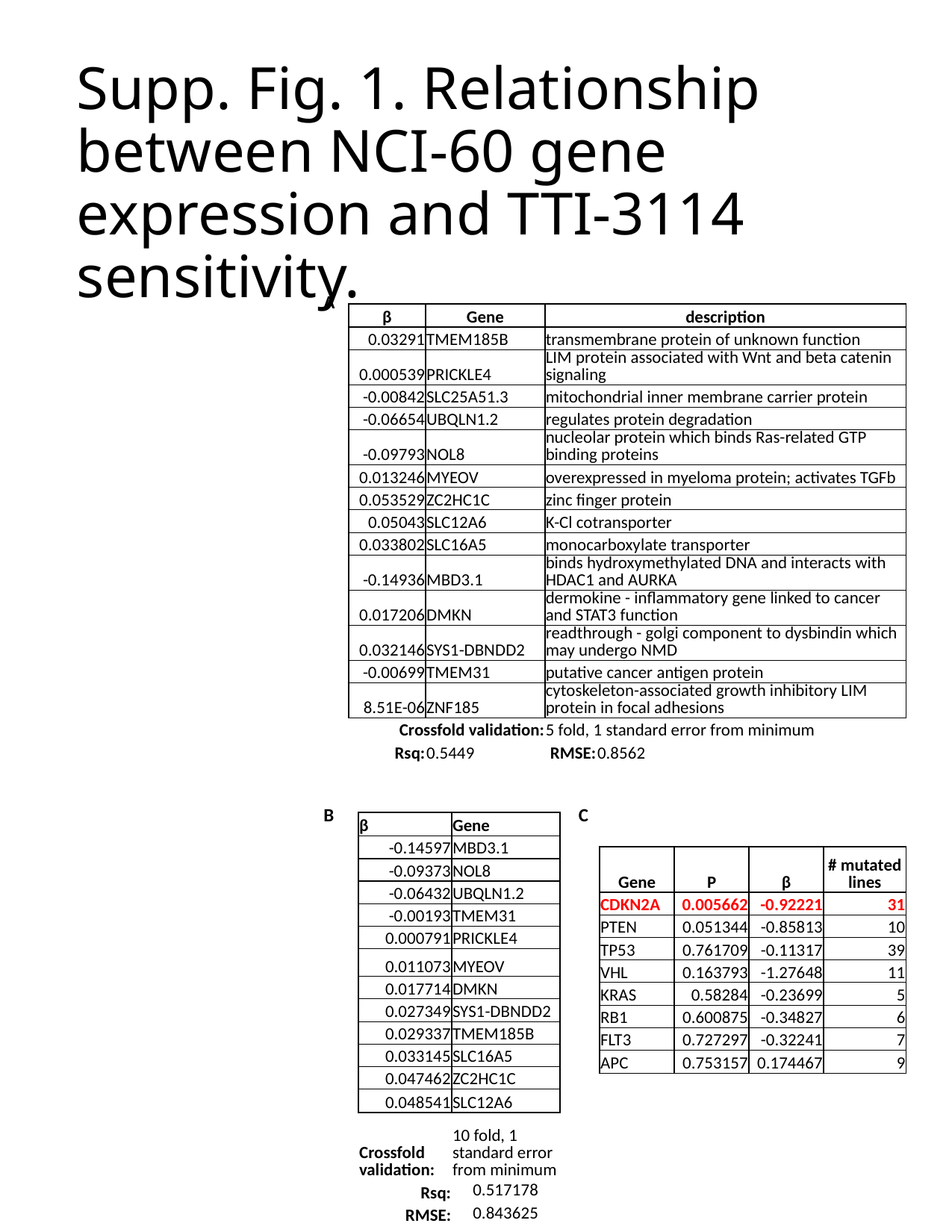

# Supp. Fig. 1. Relationship between NCI-60 gene expression and TTI-3114 sensitivity.
A
| β | Gene | description | |
| --- | --- | --- | --- |
| 0.03291 | TMEM185B | transmembrane protein of unknown function | |
| 0.000539 | PRICKLE4 | LIM protein associated with Wnt and beta catenin signaling | |
| -0.00842 | SLC25A51.3 | mitochondrial inner membrane carrier protein | |
| -0.06654 | UBQLN1.2 | regulates protein degradation | |
| -0.09793 | NOL8 | nucleolar protein which binds Ras-related GTP binding proteins | |
| 0.013246 | MYEOV | overexpressed in myeloma protein; activates TGFb | |
| 0.053529 | ZC2HC1C | zinc finger protein | |
| 0.05043 | SLC12A6 | K-Cl cotransporter | |
| 0.033802 | SLC16A5 | monocarboxylate transporter | |
| -0.14936 | MBD3.1 | binds hydroxymethylated DNA and interacts with HDAC1 and AURKA | |
| 0.017206 | DMKN | dermokine - inflammatory gene linked to cancer and STAT3 function | |
| 0.032146 | SYS1-DBNDD2 | readthrough - golgi component to dysbindin which may undergo NMD | |
| -0.00699 | TMEM31 | putative cancer antigen protein | |
| 8.51E-06 | ZNF185 | cytoskeleton-associated growth inhibitory LIM protein in focal adhesions | |
| Crossfold validation: | | 5 fold, 1 standard error from minimum | |
| Rsq: | 0.5449 | RMSE: | 0.8562 |
B
C
| β | Gene |
| --- | --- |
| -0.14597 | MBD3.1 |
| -0.09373 | NOL8 |
| -0.06432 | UBQLN1.2 |
| -0.00193 | TMEM31 |
| 0.000791 | PRICKLE4 |
| 0.011073 | MYEOV |
| 0.017714 | DMKN |
| 0.027349 | SYS1-DBNDD2 |
| 0.029337 | TMEM185B |
| 0.033145 | SLC16A5 |
| 0.047462 | ZC2HC1C |
| 0.048541 | SLC12A6 |
| Crossfold validation: | 10 fold, 1 standard error from minimum |
| Rsq: | 0.517178 |
| RMSE: | 0.843625 |
| Gene | P | β | # mutated lines |
| --- | --- | --- | --- |
| CDKN2A | 0.005662 | -0.92221 | 31 |
| PTEN | 0.051344 | -0.85813 | 10 |
| TP53 | 0.761709 | -0.11317 | 39 |
| VHL | 0.163793 | -1.27648 | 11 |
| KRAS | 0.58284 | -0.23699 | 5 |
| RB1 | 0.600875 | -0.34827 | 6 |
| FLT3 | 0.727297 | -0.32241 | 7 |
| APC | 0.753157 | 0.174467 | 9 |

## Slide 2
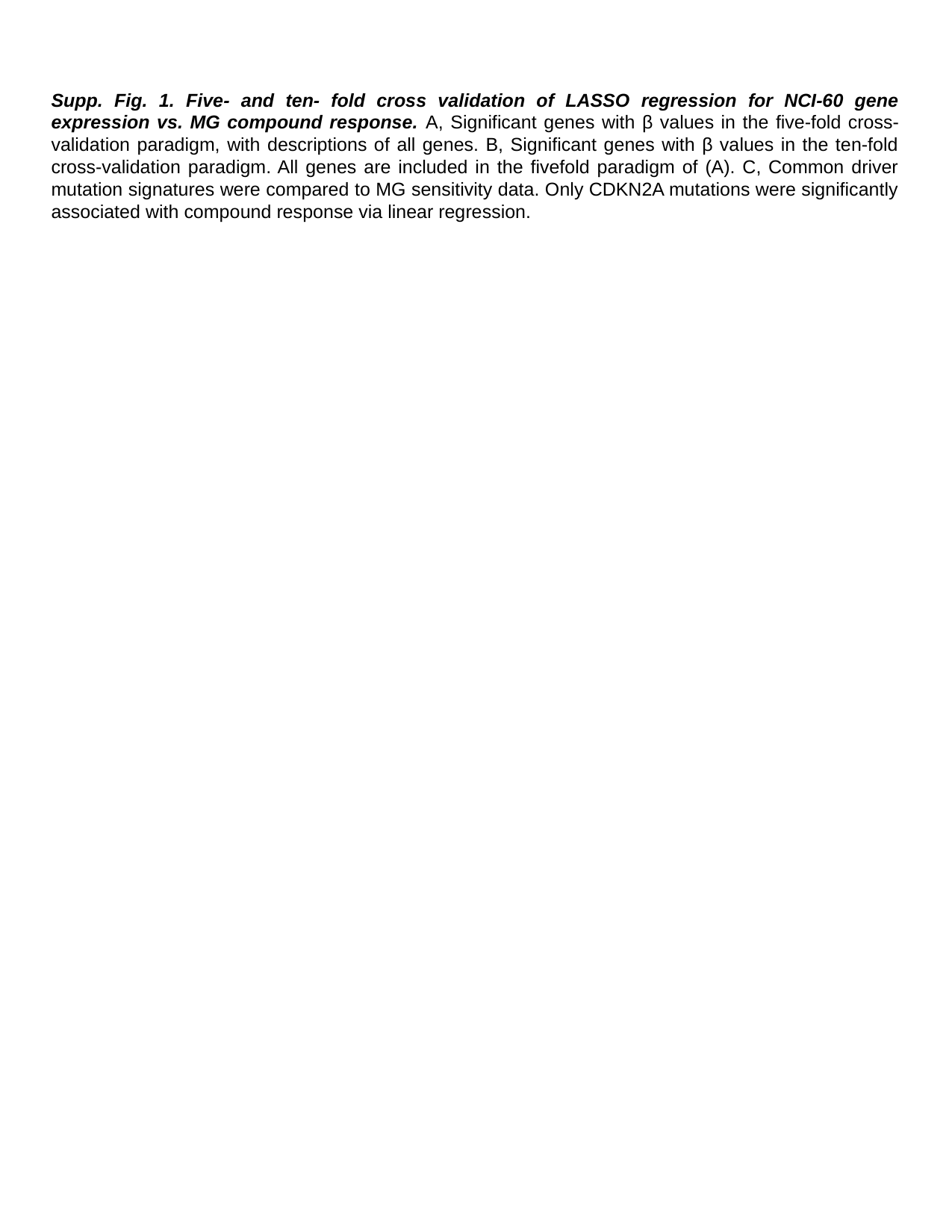

Supp. Fig. 1. Five- and ten- fold cross validation of LASSO regression for NCI-60 gene expression vs. MG compound response. A, Significant genes with β values in the five-fold cross-validation paradigm, with descriptions of all genes. B, Significant genes with β values in the ten-fold cross-validation paradigm. All genes are included in the fivefold paradigm of (A). C, Common driver mutation signatures were compared to MG sensitivity data. Only CDKN2A mutations were significantly associated with compound response via linear regression.

## Slide 3
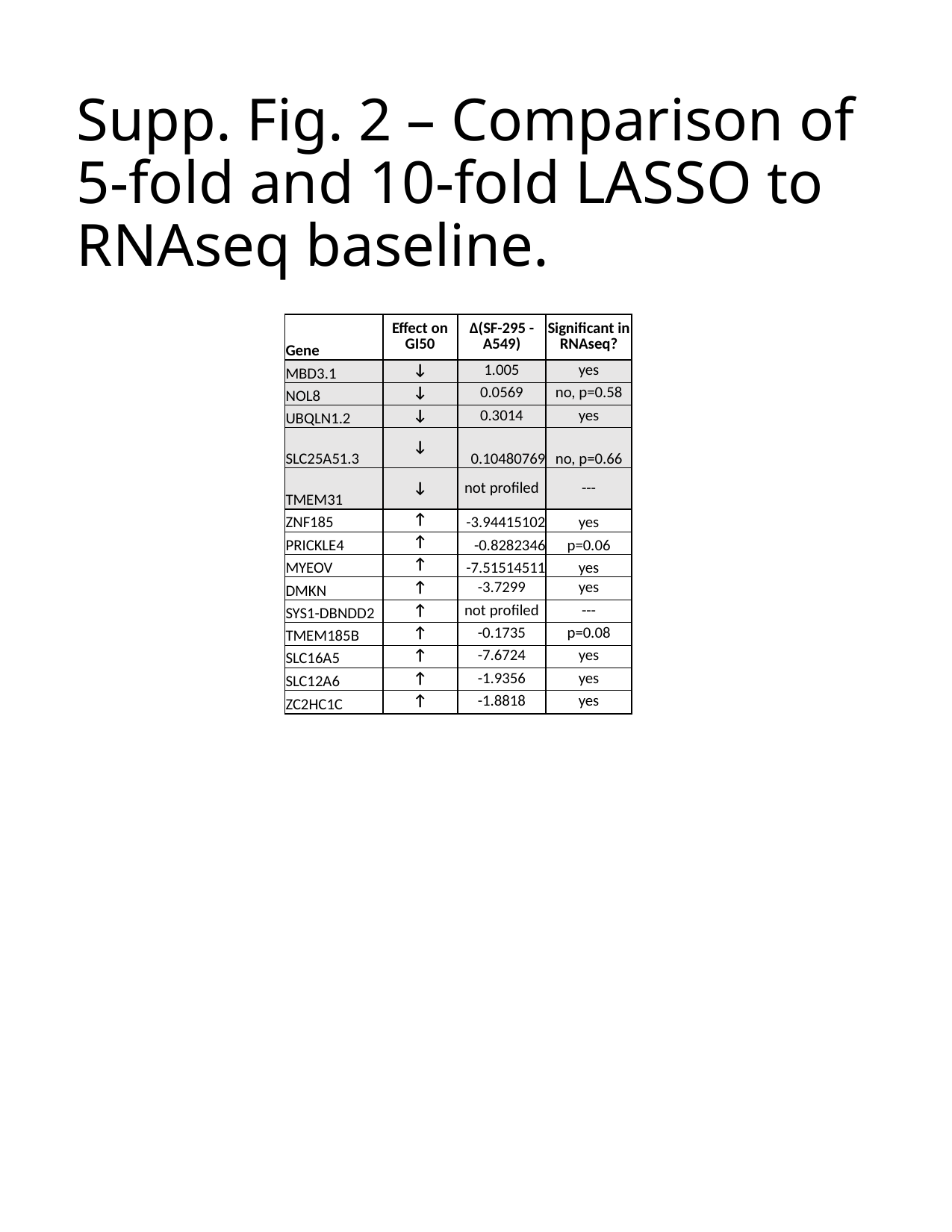

# Supp. Fig. 2 – Comparison of 5-fold and 10-fold LASSO to RNAseq baseline.
| Gene | Effect on GI50 | Δ(SF-295 - A549) | Significant in RNAseq? |
| --- | --- | --- | --- |
| MBD3.1 | ↓ | 1.005 | yes |
| NOL8 | ↓ | 0.0569 | no, p=0.58 |
| UBQLN1.2 | ↓ | 0.3014 | yes |
| SLC25A51.3 | ↓ | 0.10480769 | no, p=0.66 |
| TMEM31 | ↓ | not profiled | --- |
| ZNF185 | ↑ | -3.94415102 | yes |
| PRICKLE4 | ↑ | -0.8282346 | p=0.06 |
| MYEOV | ↑ | -7.51514511 | yes |
| DMKN | ↑ | -3.7299 | yes |
| SYS1-DBNDD2 | ↑ | not profiled | --- |
| TMEM185B | ↑ | -0.1735 | p=0.08 |
| SLC16A5 | ↑ | -7.6724 | yes |
| SLC12A6 | ↑ | -1.9356 | yes |
| ZC2HC1C | ↑ | -1.8818 | yes |

## Slide 4
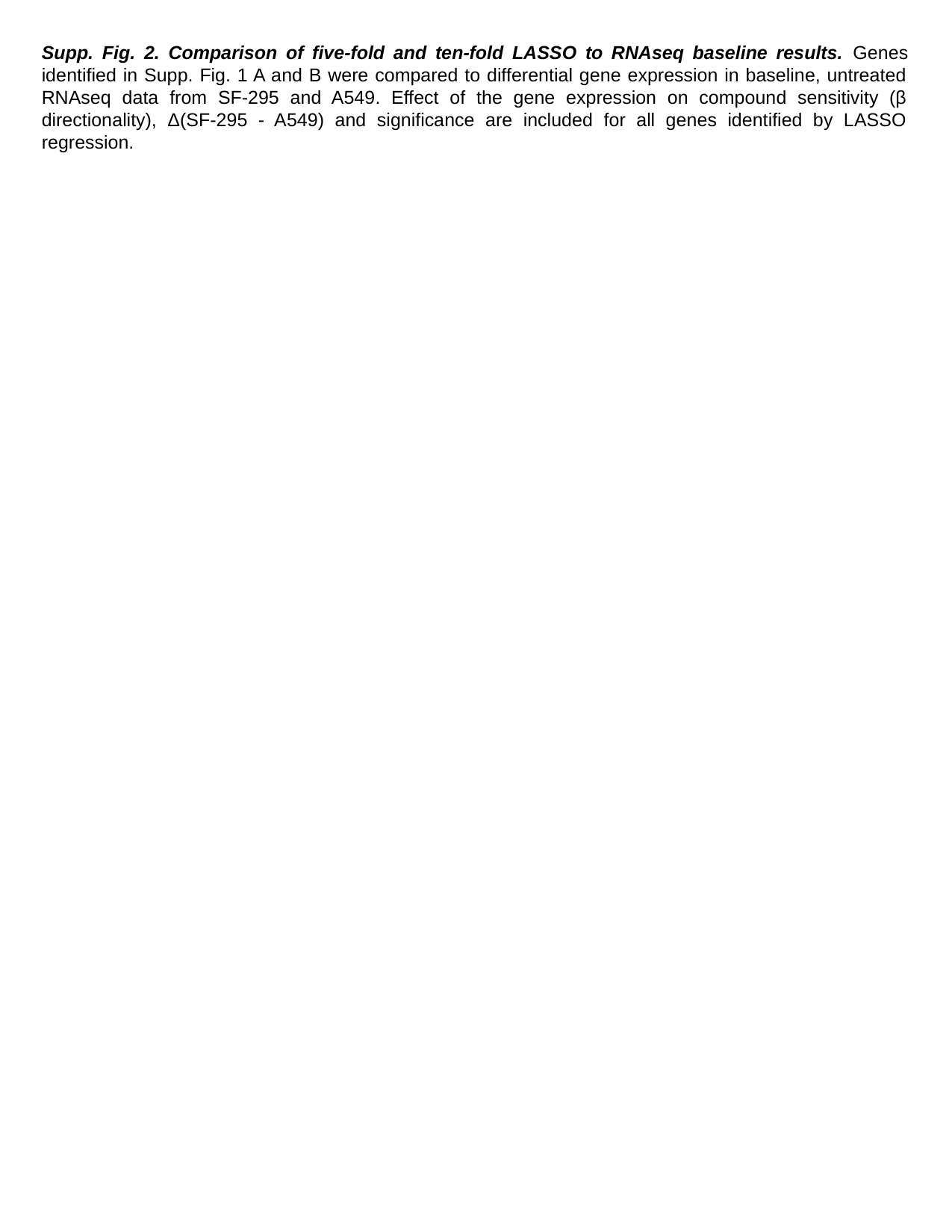

Supp. Fig. 2. Comparison of five-fold and ten-fold LASSO to RNAseq baseline results. Genes identified in Supp. Fig. 1 A and B were compared to differential gene expression in baseline, untreated RNAseq data from SF-295 and A549. Effect of the gene expression on compound sensitivity (β directionality), Δ(SF-295 - A549) and significance are included for all genes identified by LASSO regression.

## Slide 5
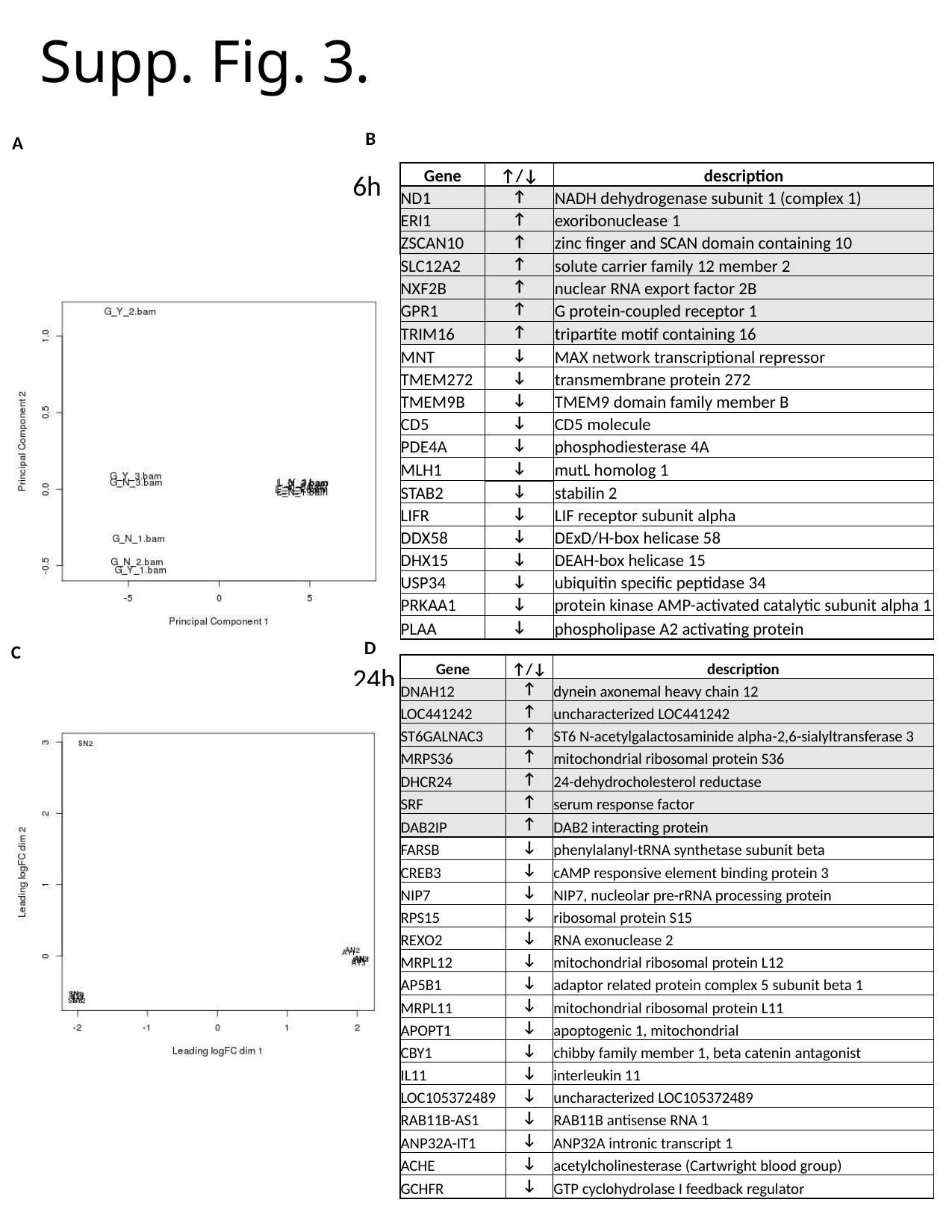

# Supp. Fig. 3.
B
A
6h
| Gene | ↑/↓ | description |
| --- | --- | --- |
| ND1 | ↑ | NADH dehydrogenase subunit 1 (complex 1) |
| ERI1 | ↑ | exoribonuclease 1 |
| ZSCAN10 | ↑ | zinc finger and SCAN domain containing 10 |
| SLC12A2 | ↑ | solute carrier family 12 member 2 |
| NXF2B | ↑ | nuclear RNA export factor 2B |
| GPR1 | ↑ | G protein-coupled receptor 1 |
| TRIM16 | ↑ | tripartite motif containing 16 |
| MNT | ↓ | MAX network transcriptional repressor |
| TMEM272 | ↓ | transmembrane protein 272 |
| TMEM9B | ↓ | TMEM9 domain family member B |
| CD5 | ↓ | CD5 molecule |
| PDE4A | ↓ | phosphodiesterase 4A |
| MLH1 | ↓ | mutL homolog 1 |
| STAB2 | ↓ | stabilin 2 |
| LIFR | ↓ | LIF receptor subunit alpha |
| DDX58 | ↓ | DExD/H-box helicase 58 |
| DHX15 | ↓ | DEAH-box helicase 15 |
| USP34 | ↓ | ubiquitin specific peptidase 34 |
| PRKAA1 | ↓ | protein kinase AMP-activated catalytic subunit alpha 1 |
| PLAA | ↓ | phospholipase A2 activating protein |
D
C
24h
| Gene | ↑/↓ | description |
| --- | --- | --- |
| DNAH12 | ↑ | dynein axonemal heavy chain 12 |
| LOC441242 | ↑ | uncharacterized LOC441242 |
| ST6GALNAC3 | ↑ | ST6 N-acetylgalactosaminide alpha-2,6-sialyltransferase 3 |
| MRPS36 | ↑ | mitochondrial ribosomal protein S36 |
| DHCR24 | ↑ | 24-dehydrocholesterol reductase |
| SRF | ↑ | serum response factor |
| DAB2IP | ↑ | DAB2 interacting protein |
| FARSB | ↓ | phenylalanyl-tRNA synthetase subunit beta |
| CREB3 | ↓ | cAMP responsive element binding protein 3 |
| NIP7 | ↓ | NIP7, nucleolar pre-rRNA processing protein |
| RPS15 | ↓ | ribosomal protein S15 |
| REXO2 | ↓ | RNA exonuclease 2 |
| MRPL12 | ↓ | mitochondrial ribosomal protein L12 |
| AP5B1 | ↓ | adaptor related protein complex 5 subunit beta 1 |
| MRPL11 | ↓ | mitochondrial ribosomal protein L11 |
| APOPT1 | ↓ | apoptogenic 1, mitochondrial |
| CBY1 | ↓ | chibby family member 1, beta catenin antagonist |
| IL11 | ↓ | interleukin 11 |
| LOC105372489 | ↓ | uncharacterized LOC105372489 |
| RAB11B-AS1 | ↓ | RAB11B antisense RNA 1 |
| ANP32A-IT1 | ↓ | ANP32A intronic transcript 1 |
| ACHE | ↓ | acetylcholinesterase (Cartwright blood group) |
| GCHFR | ↓ | GTP cyclohydrolase I feedback regulator |

## Slide 6
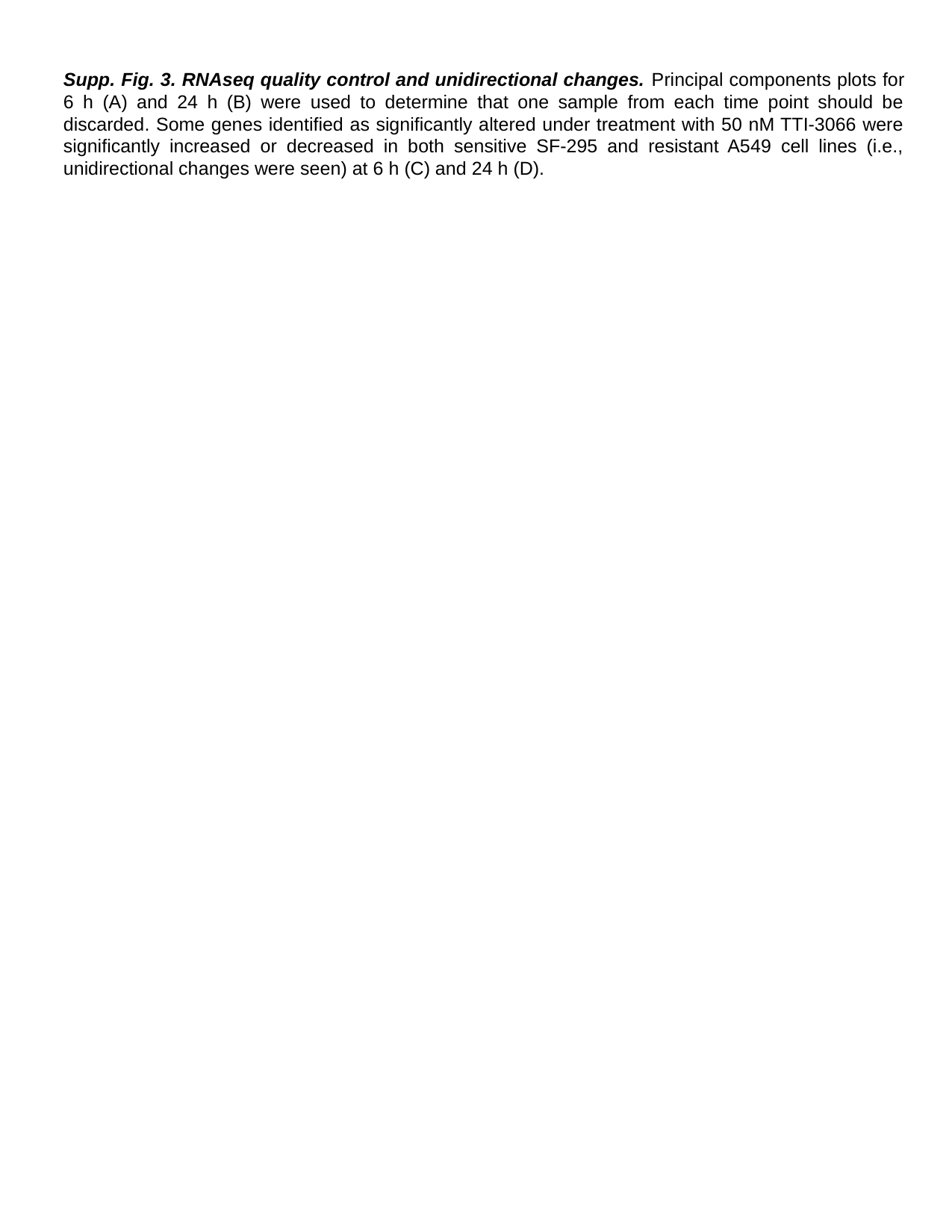

Supp. Fig. 3. RNAseq quality control and unidirectional changes. Principal components plots for 6 h (A) and 24 h (B) were used to determine that one sample from each time point should be discarded. Some genes identified as significantly altered under treatment with 50 nM TTI-3066 were significantly increased or decreased in both sensitive SF-295 and resistant A549 cell lines (i.e., unidirectional changes were seen) at 6 h (C) and 24 h (D).

## Slide 7
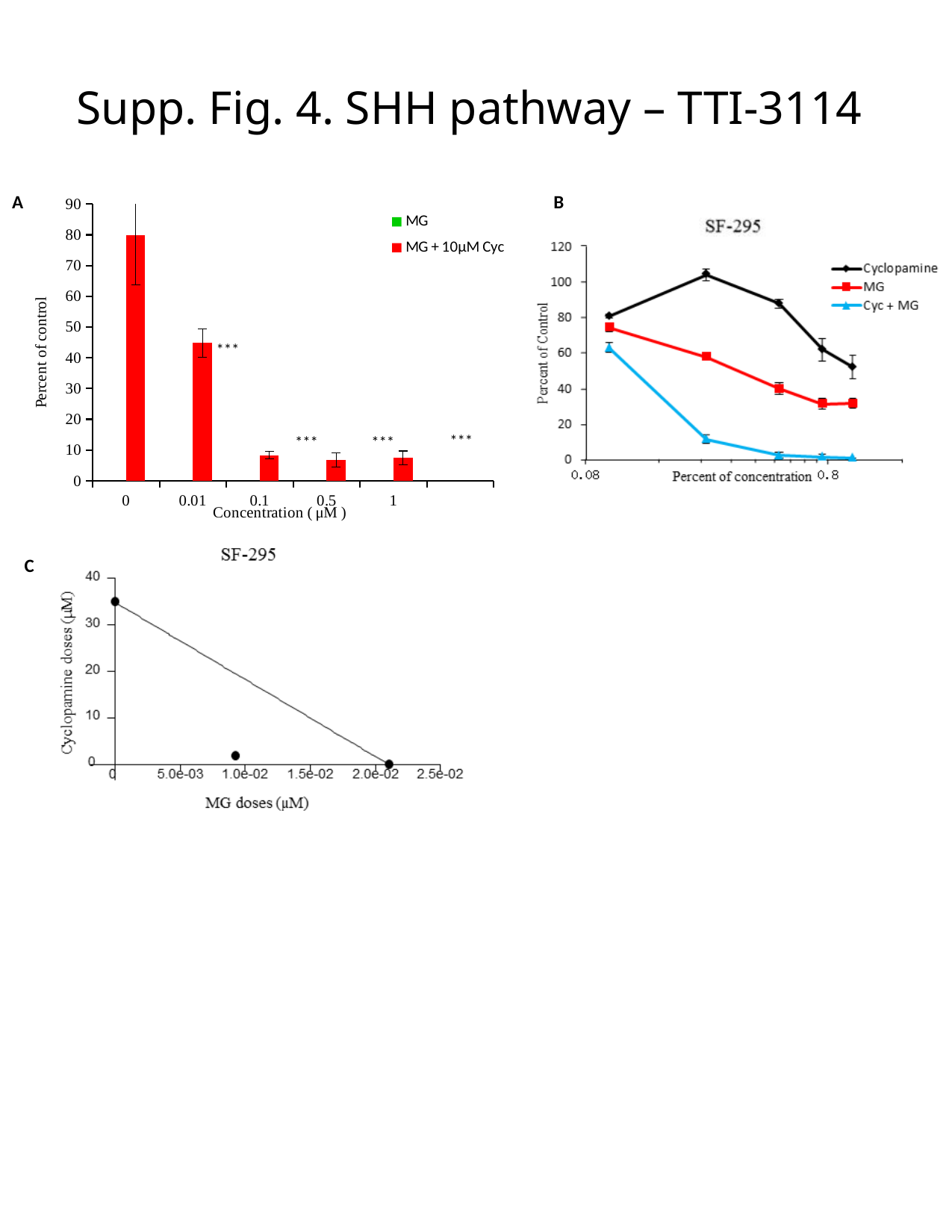

# Supp. Fig. 4. SHH pathway – TTI-3114
### Chart
| Category | | MG + 10μM Cyc |
|---|---|---|
| 0 | 100.0 | 79.8377522754254 |
| 0.01 | 92.87729196050776 | 44.783010156971365 |
| 0.1 | 71.36812411847673 | 8.310249307479225 |
| 0.5 | 41.46685472496475 | 6.740535549399813 |
| 1 | 31.946403385049365 | 7.386888273314865 |B
A
***
***
***
***
C

## Slide 8
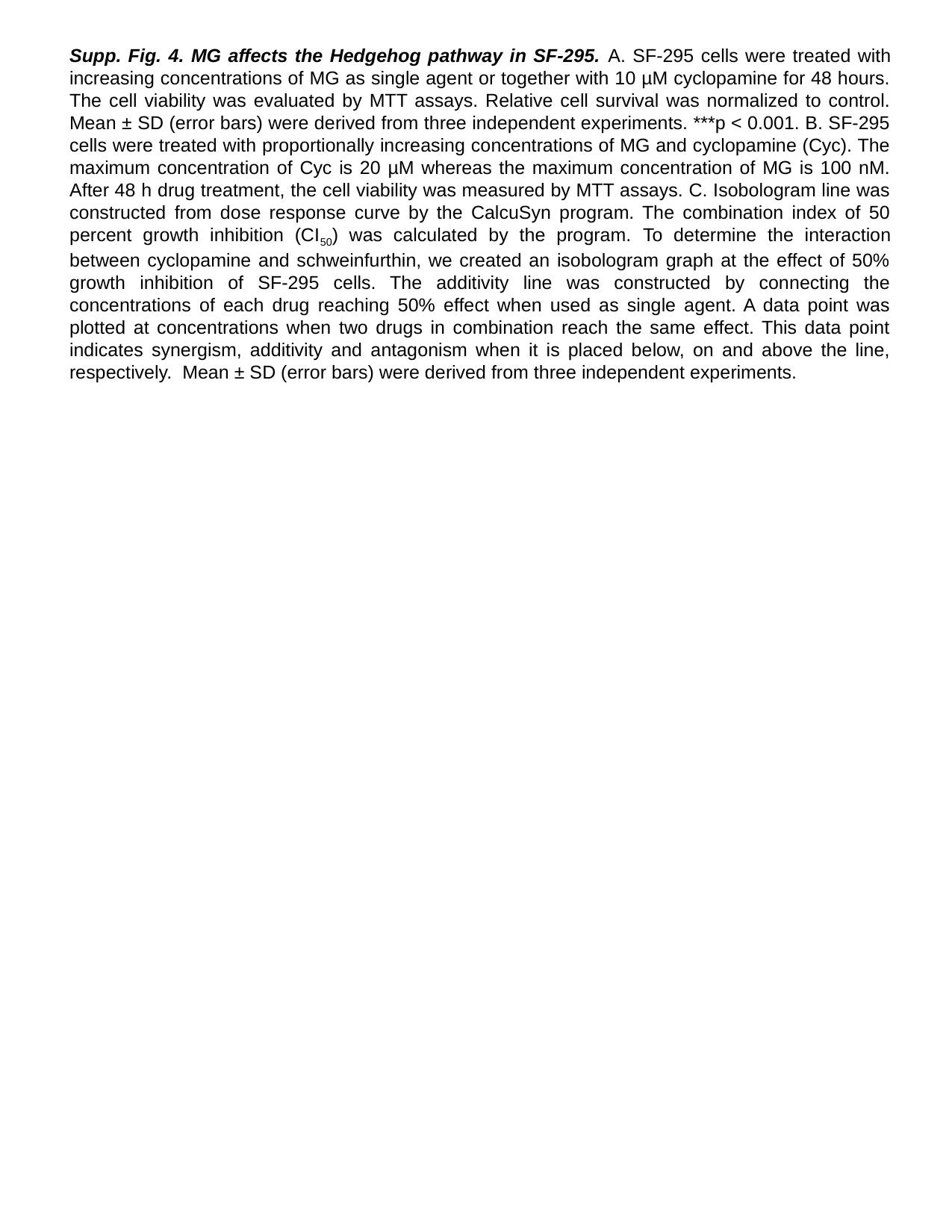

Supp. Fig. 4. MG affects the Hedgehog pathway in SF-295. A. SF-295 cells were treated with increasing concentrations of MG as single agent or together with 10 µM cyclopamine for 48 hours. The cell viability was evaluated by MTT assays. Relative cell survival was normalized to control. Mean ± SD (error bars) were derived from three independent experiments. ***p < 0.001. B. SF-295 cells were treated with proportionally increasing concentrations of MG and cyclopamine (Cyc). The maximum concentration of Cyc is 20 µM whereas the maximum concentration of MG is 100 nM. After 48 h drug treatment, the cell viability was measured by MTT assays. C. Isobologram line was constructed from dose response curve by the CalcuSyn program. The combination index of 50 percent growth inhibition (CI50) was calculated by the program. To determine the interaction between cyclopamine and schweinfurthin, we created an isobologram graph at the effect of 50% growth inhibition of SF-295 cells. The additivity line was constructed by connecting the concentrations of each drug reaching 50% effect when used as single agent. A data point was plotted at concentrations when two drugs in combination reach the same effect. This data point indicates synergism, additivity and antagonism when it is placed below, on and above the line, respectively. Mean ± SD (error bars) were derived from three independent experiments.

## Slide 9
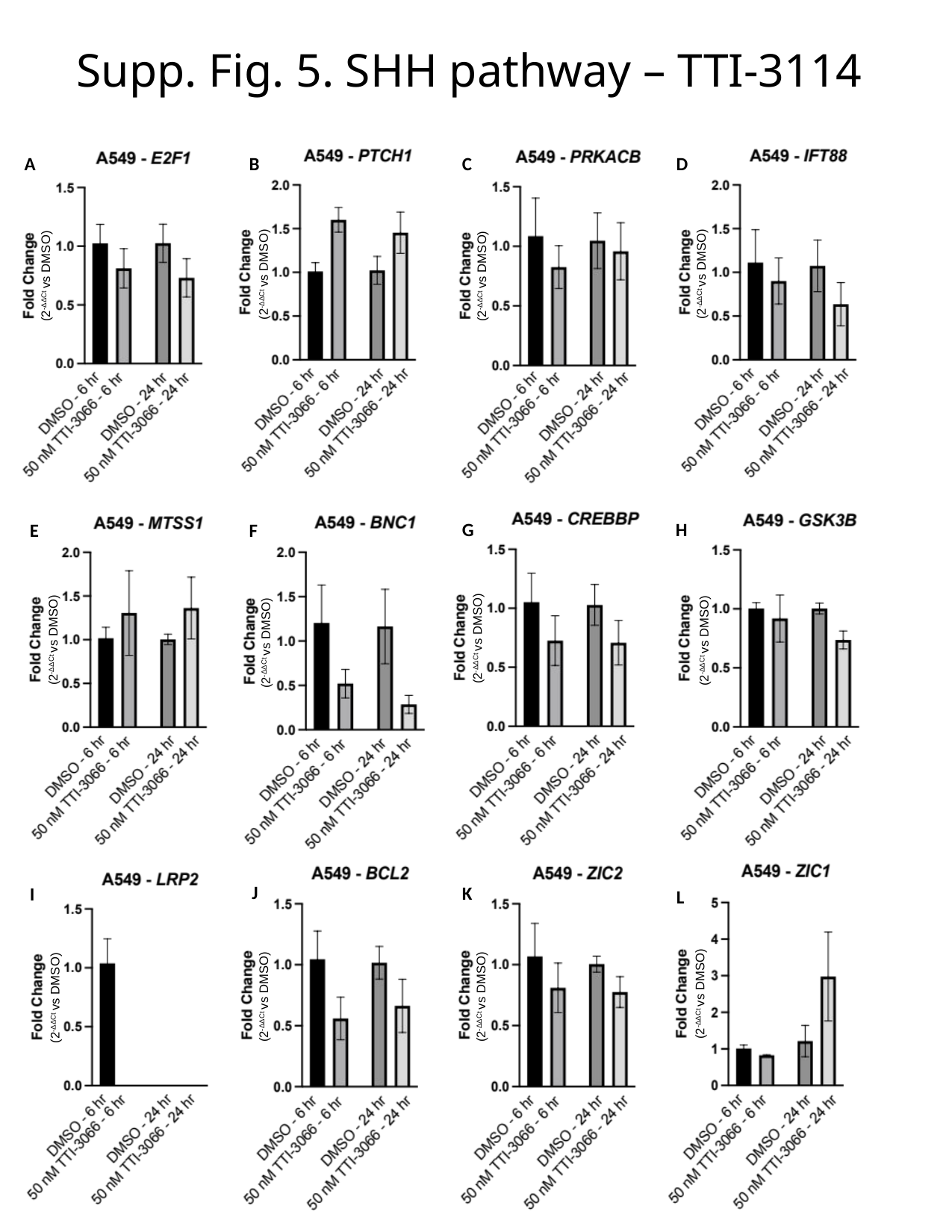

Supp. Fig. 5. SHH pathway – TTI-3114
(2-ΔΔCt vs DMSO)
(2-ΔΔCt vs DMSO)
(2-ΔΔCt vs DMSO)
(2-ΔΔCt vs DMSO)
D
A
B
C
(2-ΔΔCt vs DMSO)
(2-ΔΔCt vs DMSO)
(2-ΔΔCt vs DMSO)
(2-ΔΔCt vs DMSO)
G
H
F
E
(2-ΔΔCt vs DMSO)
(2-ΔΔCt vs DMSO)
(2-ΔΔCt vs DMSO)
(2-ΔΔCt vs DMSO)
J
K
I
L

## Slide 10
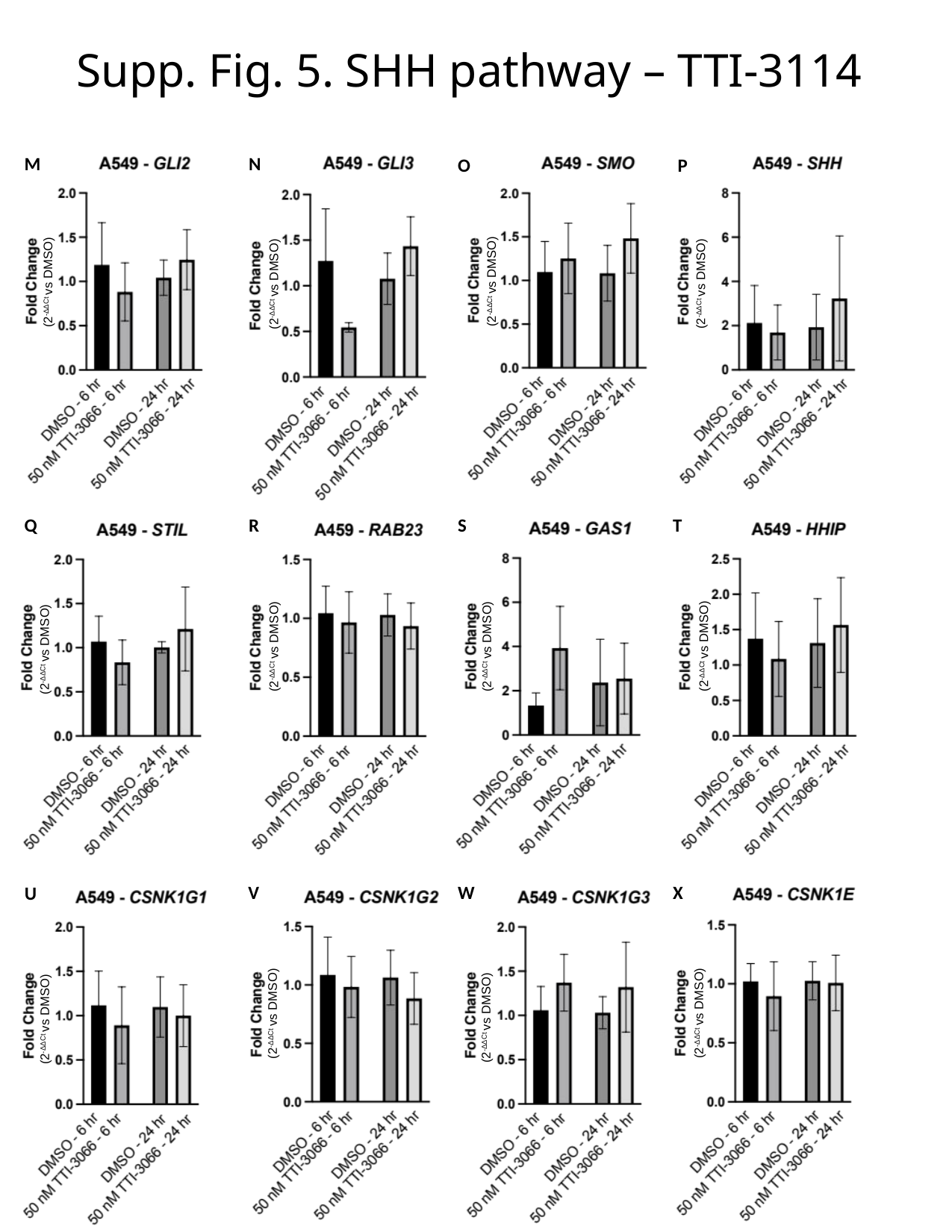

Supp. Fig. 5. SHH pathway – TTI-3114
M
N
(2-ΔΔCt vs DMSO)
(2-ΔΔCt vs DMSO)
(2-ΔΔCt vs DMSO)
P
(2-ΔΔCt vs DMSO)
O
Q
R
S
T
(2-ΔΔCt vs DMSO)
(2-ΔΔCt vs DMSO)
(2-ΔΔCt vs DMSO)
(2-ΔΔCt vs DMSO)
X
W
V
U
(2-ΔΔCt vs DMSO)
(2-ΔΔCt vs DMSO)
(2-ΔΔCt vs DMSO)
(2-ΔΔCt vs DMSO)

## Slide 11
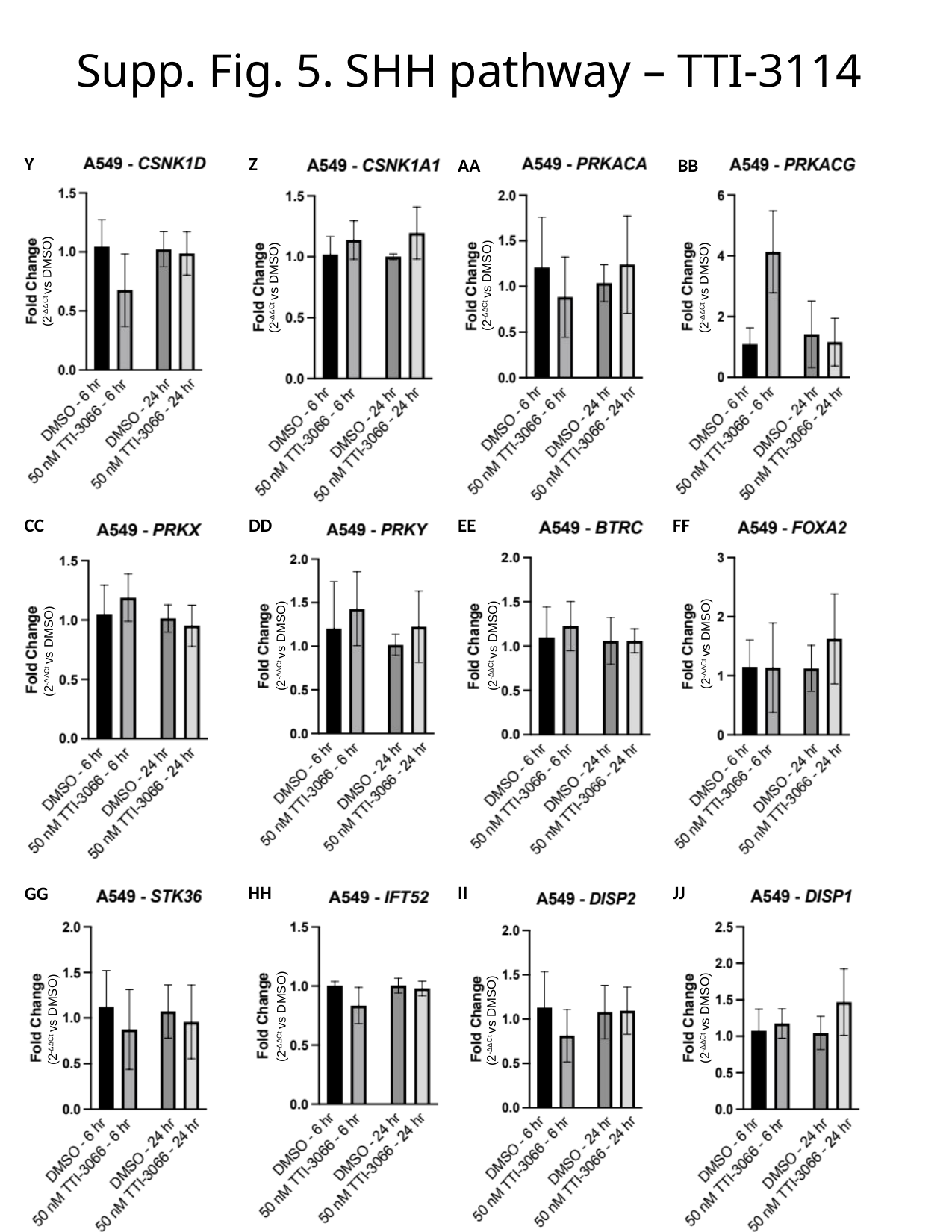

Supp. Fig. 5. SHH pathway – TTI-3114
Y
Z
(2-ΔΔCt vs DMSO)
(2-ΔΔCt vs DMSO)
BB
AA
(2-ΔΔCt vs DMSO)
(2-ΔΔCt vs DMSO)
CC
DD
EE
FF
(2-ΔΔCt vs DMSO)
(2-ΔΔCt vs DMSO)
(2-ΔΔCt vs DMSO)
(2-ΔΔCt vs DMSO)
JJ
II
HH
GG
(2-ΔΔCt vs DMSO)
(2-ΔΔCt vs DMSO)
(2-ΔΔCt vs DMSO)
(2-ΔΔCt vs DMSO)

## Slide 12
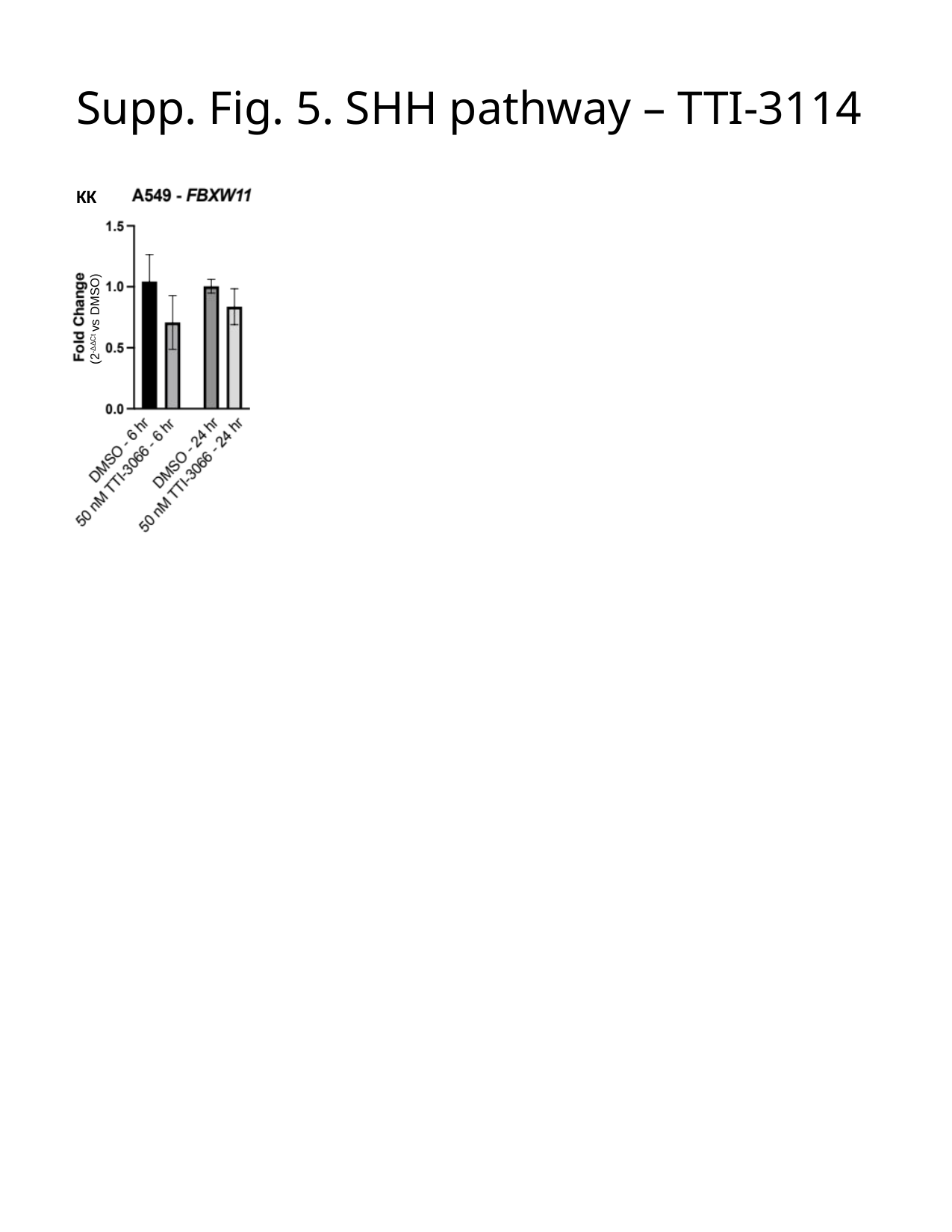

Supp. Fig. 5. SHH pathway – TTI-3114
KK
(2-ΔΔCt vs DMSO)

## Slide 13
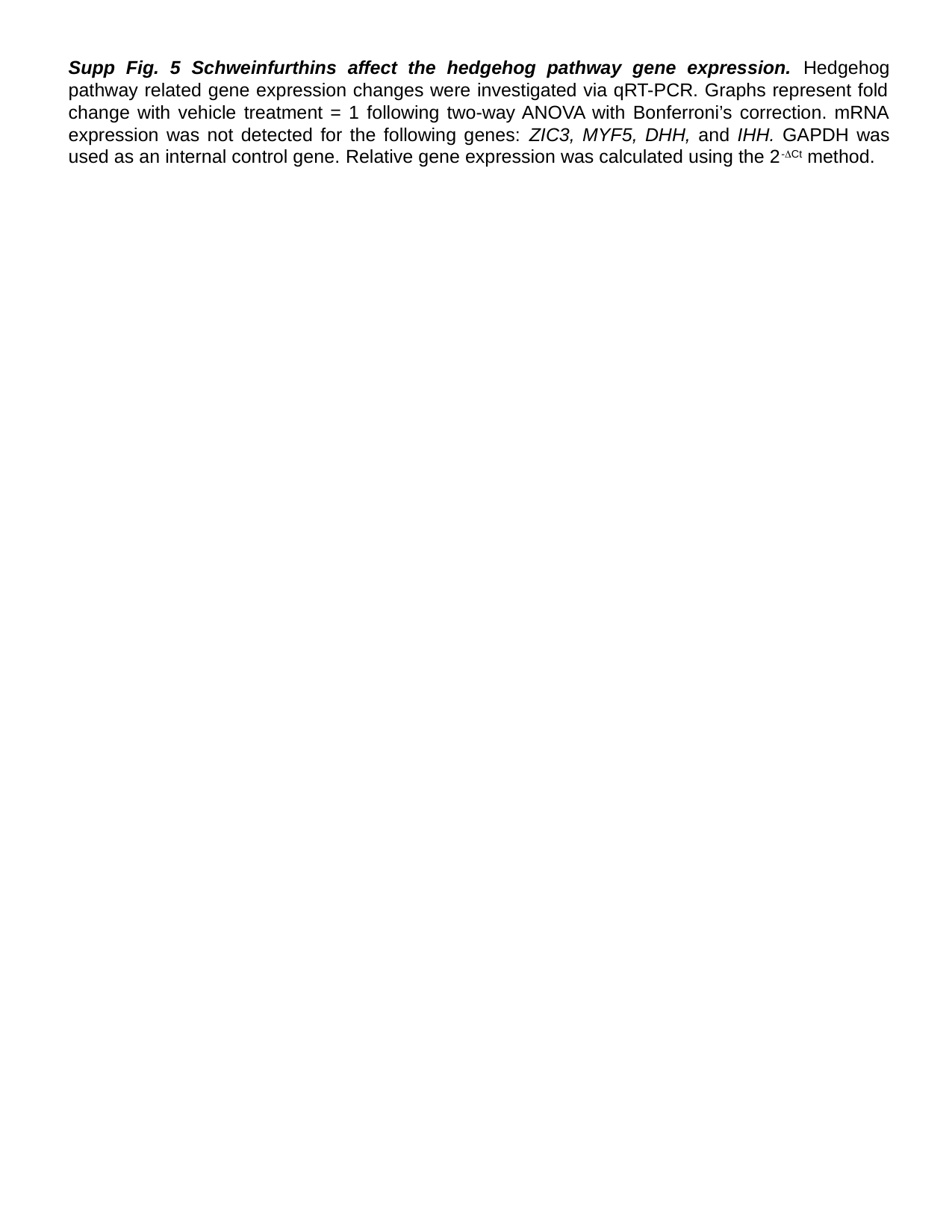

Supp Fig. 5 Schweinfurthins affect the hedgehog pathway gene expression. Hedgehog pathway related gene expression changes were investigated via qRT-PCR. Graphs represent fold change with vehicle treatment = 1 following two-way ANOVA with Bonferroni’s correction. mRNA expression was not detected for the following genes: ZIC3, MYF5, DHH, and IHH. GAPDH was used as an internal control gene. Relative gene expression was calculated using the 2-ΔCt method.

## Slide 14
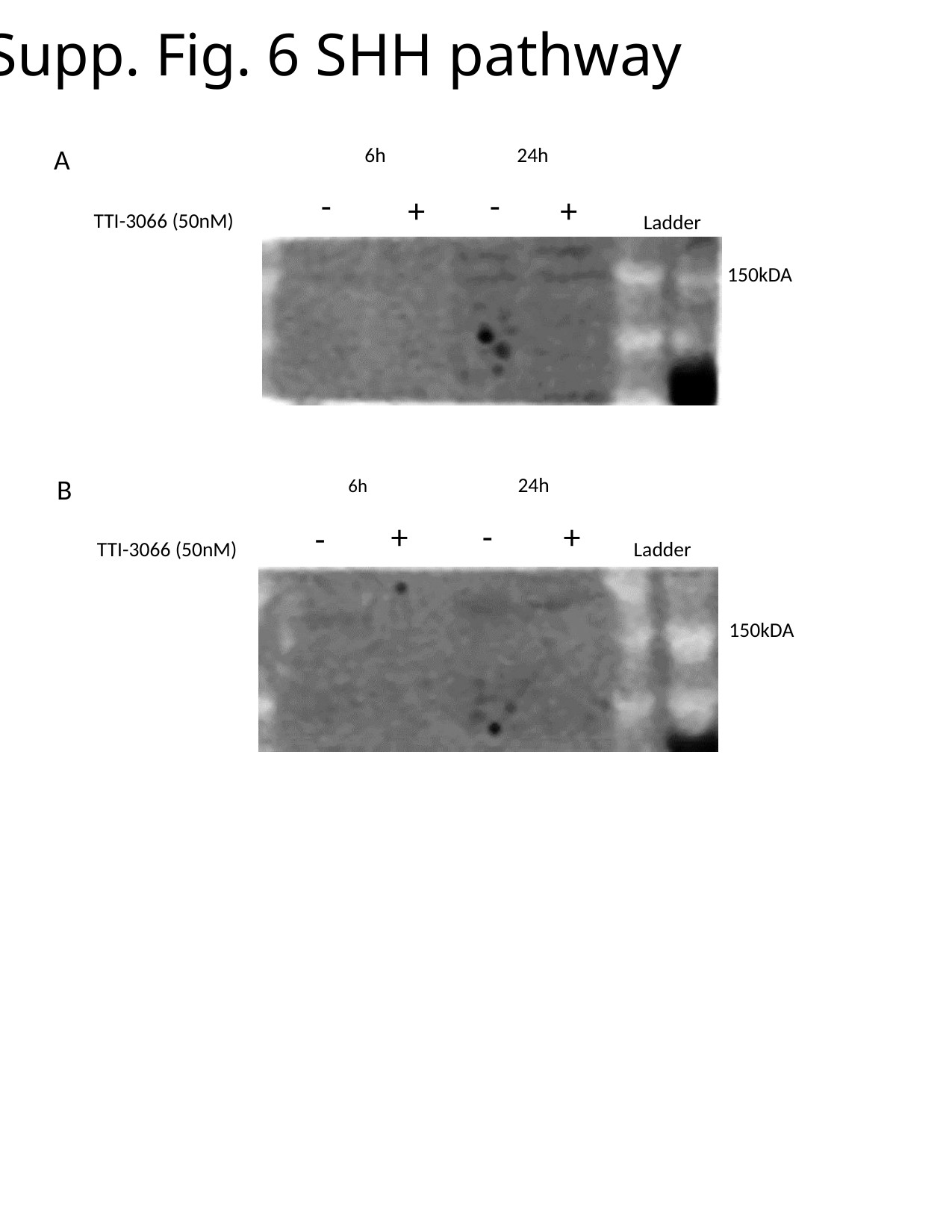

Supp. Fig. 6 SHH pathway
A
24h
6h
-
-
+
+
TTI-3066 (50nM)
Ladder
150kDA
24h
B
6h
+
-
+
-
TTI-3066 (50nM)
Ladder
150kDA

## Slide 15
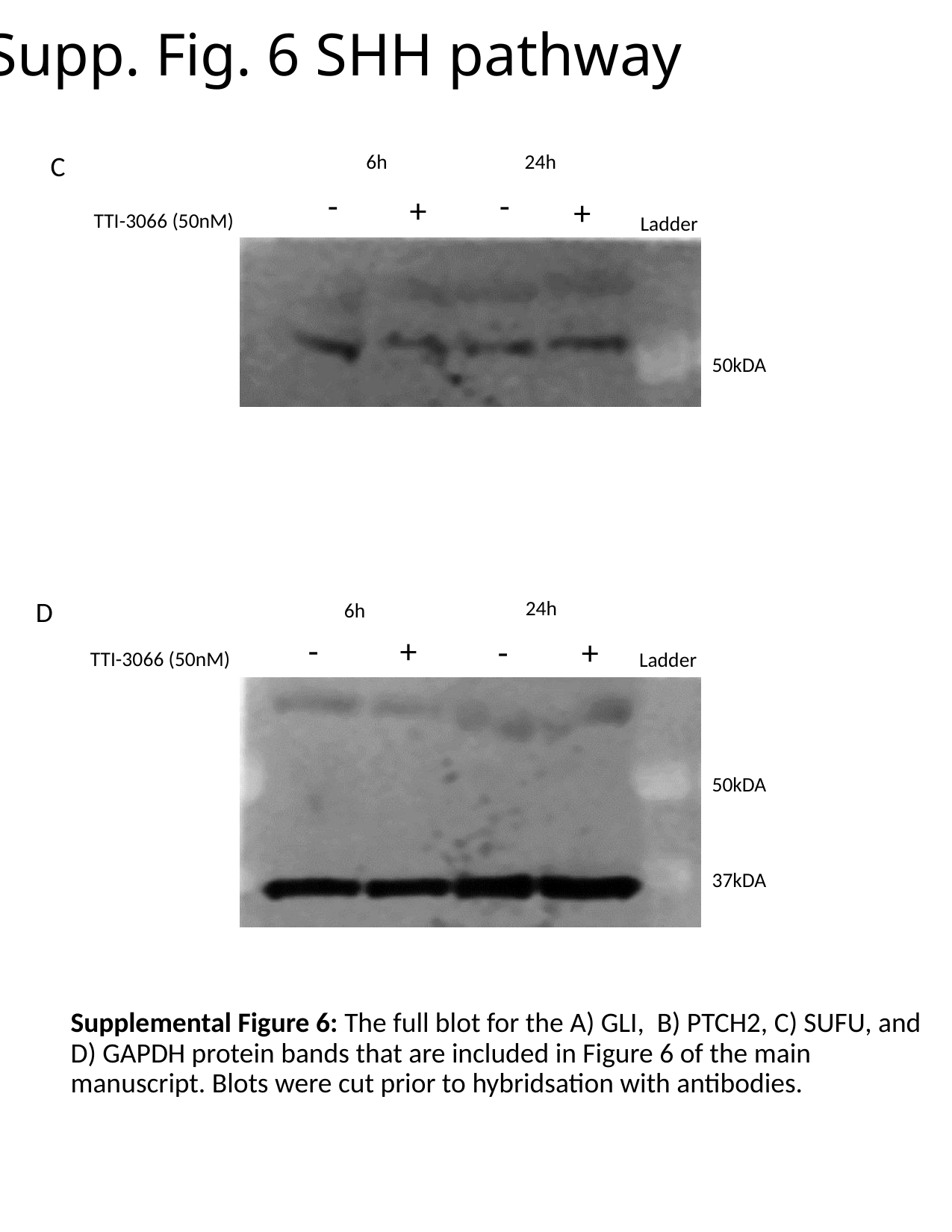

Supp. Fig. 6 SHH pathway
C
6h
24h
-
-
+
+
TTI-3066 (50nM)
Ladder
50kDA
D
24h
6h
-
+
-
+
TTI-3066 (50nM)
Ladder
50kDA
37kDA
Supplemental Figure 6: The full blot for the A) GLI, B) PTCH2, C) SUFU, and D) GAPDH protein bands that are included in Figure 6 of the main manuscript. Blots were cut prior to hybridsation with antibodies.

## Slide 16
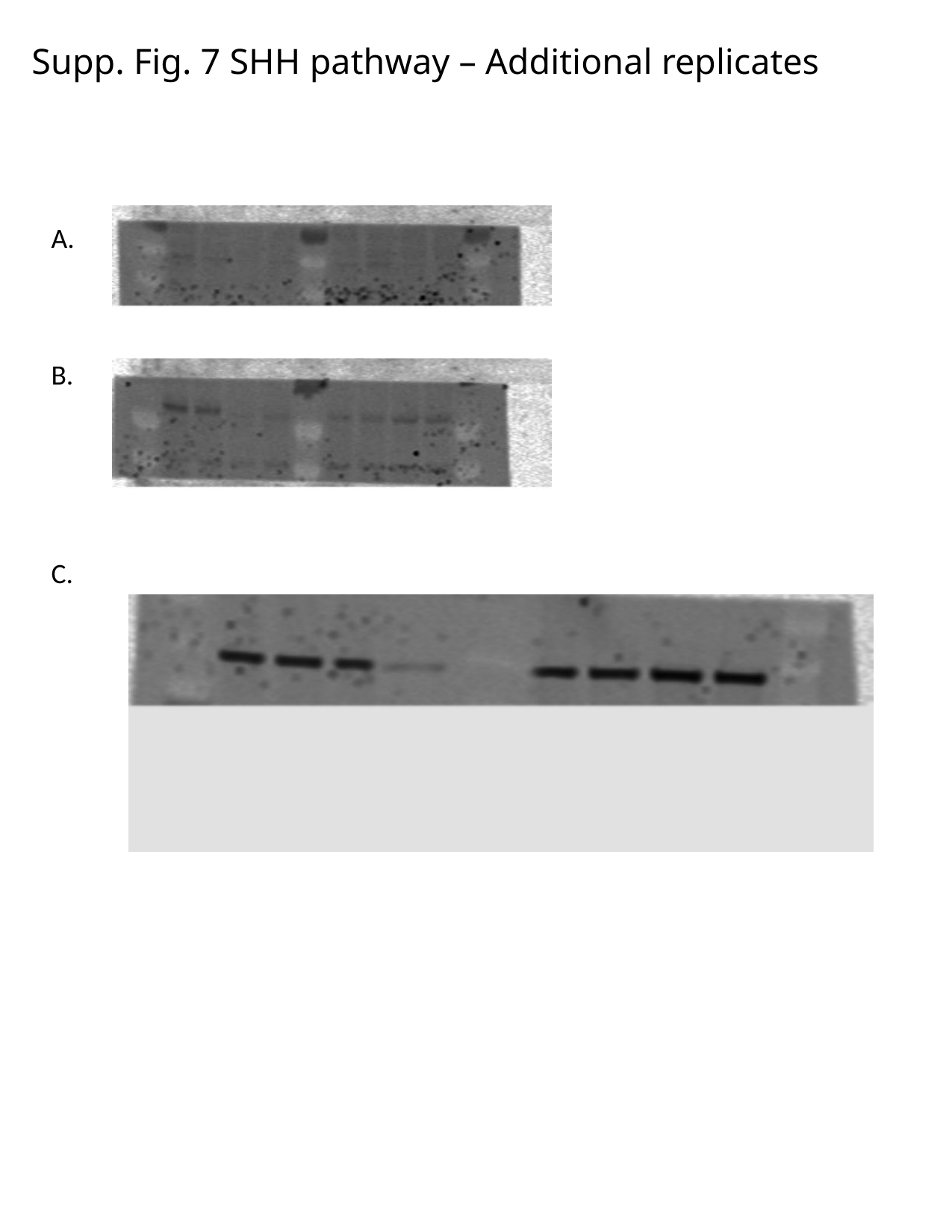

# Supp. Fig. 7 SHH pathway – Additional replicates
A.
B.
C.

## Slide 17
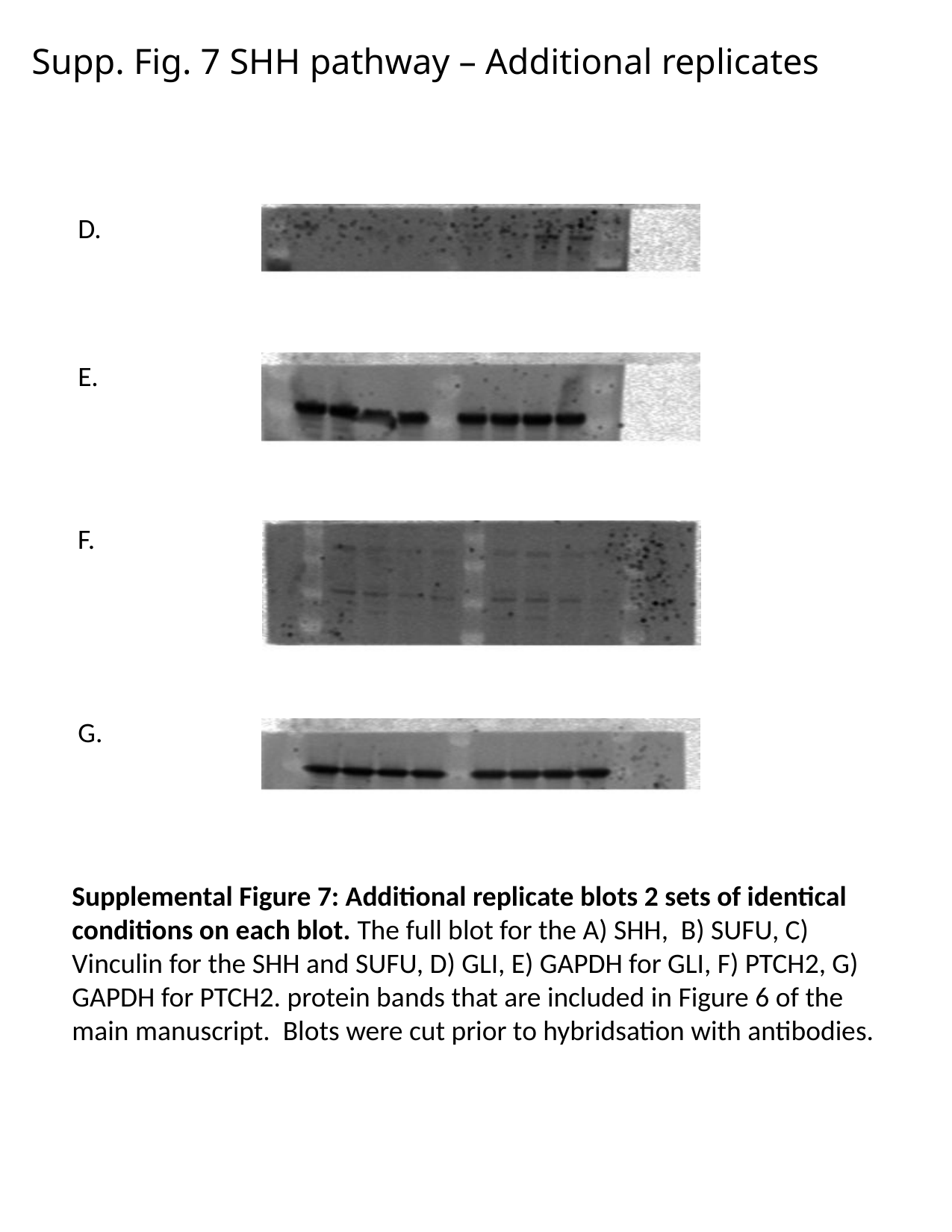

# Supp. Fig. 7 SHH pathway – Additional replicates
D.
E.
F.
G.
Supplemental Figure 7: Additional replicate blots 2 sets of identical conditions on each blot. The full blot for the A) SHH, B) SUFU, C) Vinculin for the SHH and SUFU, D) GLI, E) GAPDH for GLI, F) PTCH2, G) GAPDH for PTCH2. protein bands that are included in Figure 6 of the main manuscript. Blots were cut prior to hybridsation with antibodies.
